# The pleiotropic landscape of rare variant associations with multiple cancers in large biobanks

**DOI:** 10.1016/j.xhgg.2026.100662

**Published:** 2026-08-20

**Authors:** Austin Hammermeister Suger, Tabitha A. Harrison, Jiachen Zhang, Michael C. Wu, Burcu F. Darst, Sara Lindström

**Affiliations:** 1 Department of Epidemiology, University of Washington School of Public Health, 3980 15th Ave. NE, Seattle, WA 98195, USA; 2 Institute for Public Health Genetics, University of Washington School of Public Health, 1705 NE Pacific St., Seattle, WA 98195, USA; 3 Public Health Sciences Division, Fred Hutchinson Cancer Center, 1100 Fairview Ave. N, Seattle, WA 98109, USA

**Keywords:** rare variants, pleiotropy, exome sequencing, multiple cancers, biobanks

## Abstract

Previous association studies between germline rare genetic variation and cancer risk have primarily examined a limited number of cancers in clinical samples, often with participants predominantly of European genetic ancestry. We conducted exome-wide rare variant association analyses across 70+ cancer types using data from more than 729,000 participants from the UK Biobank (UKB) and *All of Us* (AoU) Research Program cohorts. We used generalized linear mixed models to screen for cancer pleiotropic effects by conducting gene-based and single-variant tests of predicted loss of function (pLoF) and missense variants and six groups of cancer defined by biological and etiological similarities. We then assessed significant genes for associations with 27 individual cancer types that had data for at least 500 affected individuals. Of the 33 potential pleiotropic genes identified, 16 consistently showed significant associations across ≥3 individual cancer types. For example, the presence of at least one *CHEK2* pLoF variant was associated with increased odds of diagnosis with 14 different cancers (odds ratio [OR] range: 1.34–3.21). Similarly, the presence of at least one *RTEL1* missense variant was associated with lower odds of diagnosis with 9 cancers (OR range: 0.67–0.88). Our results expand our knowledge about these loci and point to a larger impact on overall cancer risk than previously appreciated.

## Graphical abstract

**Figure:**
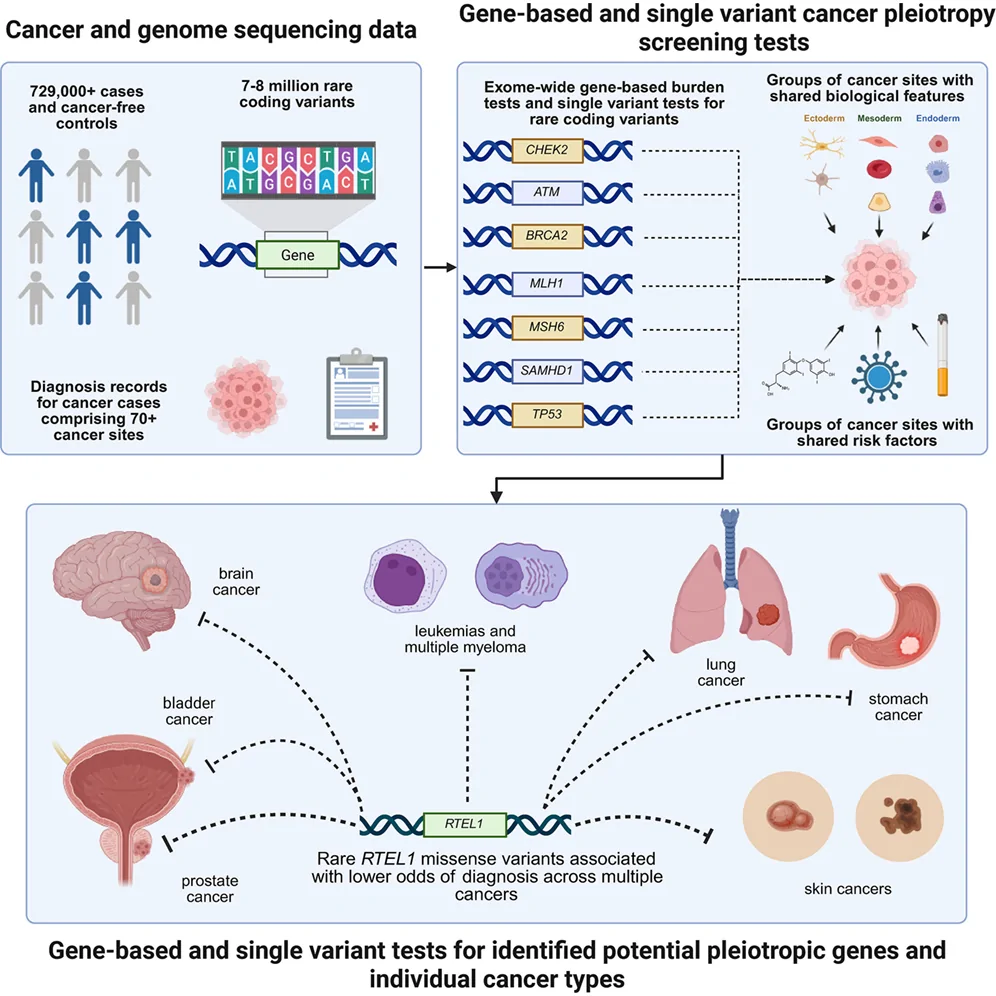

## Teaser

Researchers used data from the UK Biobank and All of Us Research Program to identify genes where rare genetic variants are associated with cancer diagnosis across multiple cancer types. The results suggest that rare variants in certain genes may influence risk across a wider range of cancers than previously thought.

## Introduction

Cancer is a leading cause of morbidity and mortality worldwide.^1^ Characterizing the genetic basis of cancer improves our understanding of the underlying cancer biology and informs shared mechanisms across cancer types. While many known genetic risk factors for cancer are specific to a particular cancer, prior studies of common genetic variation have revealed modest genetic correlations across various cancer types,^2^^,^^3^ and recent multi-cancer genome-wide association studies (GWASs) have highlighted the pleiotropic contributions of common genetic variants to cancer risk across global populations.^4^^,^^5^^,^^6^^,^^7^ These types of multi-cancer analyses can help expand our understanding of the shared genetic basis of carcinogenesis and may inform strategies for genetic testing for hereditary multi-cancer risk.^8^^,^^9^^,^^10^

Although there has been substantial research into the genetic etiology of individual cancer types and groups of cancers, the contributions of rare variants (RVs) to the shared genetic etiology of multiple cancer types have been examined in only a handful of studies.^11^^,^^12^^,^^13^^,^^14^^,^^15^^,^^16^^,^^17^ Until recently, RV cancer studies have been limited by the need for sizable genome sequencing datasets to achieve comprehensive genome coverage and adequate statistical power. With the exception of a few recent studies,^15^^,^^16^^,^^17^ most prior studies were limited by small sample sizes or a narrow set of cancer types. In particular, no study has characterized genomic regions with pleiotropic RV associations across 20 or more cancers.

The availability of population-based biobank sequencing datasets now enables genome-wide, multi-cancer RV analyses across populations. Identifying and characterizing the contributions of RVs in these datasets allows us to expand our scope to additional cancer types often not included to help fill gaps in our understanding of the biological mechanisms involved in multiple cancer types. Further, estimating risk effects across cancer within the same datasets allowed us to compare gene-specific effects across cancers, minimizing heterogeneity due to variation in populations, sequencing technology, and computational pipelines.

One approach to identifying genomic loci where coding RVs (cRVs) exert pleiotropic effects across multiple cancers is to conduct exome-wide, gene-based tests for the diagnosis of any cancer within biologically defined cancer groups. Grouping cancers according to the germ layer of origin of the tissue from which they formed has a long history,^18^ and recent studies indicate similarities in biological processes and pathways involved in embryogenesis and tumorigenesis.^19^ Alternatively, cancer types can be grouped based on shared established risk factors, such as tobacco smoke,^20^^,^^21^ infectious agents,^22^ and endogenous and exogenous hormones,^23^^,^^24^^,^^25^^,^^26^^,^^27^^,^^28^ reflecting shared etiology. In this work, we used cancer diagnosis and genome sequencing data from 729,469 participants from the UK Biobank (UKB) and *All of Us* (AoU) cohorts to conduct an exome-wide screen for genes showing pleiotropic cRV associations across biologically and etiologically defined cancer groups. We then characterized these associations across 27 individual cancer types.

## Subjects and methods

### Study populations

We included participants with genomic, cancer diagnosis, and other core demographic data from the UKB and AoU cohorts. The UKB is a prospective cohort study of approximately 500,000 individuals aged 40–69 years at enrollment living in the United Kingdom (UK).^29^^,^^30^ The AoU is a prospective cohort study of individuals over the age of 18 living in the United States (US) with over 630,000 participants enrolled.^31^^,^^32^

### Cancer data and sample exclusions

Cancer diagnosis records in the UKB were obtained through linkage to NHS cancer registries and processed by the UKB as previously described.^29^^,^^33^ AoU cancer diagnosis records were extracted from participant electronic health record (EHR) data and processed by AoU as previously described.^34^ For both cohorts, cancer cases were defined as individuals with documented International Classification of Disease (ICD)-9 or ICD-10 codes for invasive or *in situ* cancers. The ICD code sets used to define each cancer were identical in each cohort. We included both prevalent and incident cases, excluding individuals without sequencing data or EHRs/cancer diagnosis records, those with a self-reported cancer diagnosis but no registry records or EHRs, and those with undefined or ill-defined cancer sites. For AoU, we additionally excluded participants without consent for EHR linkage. We also excluded individuals whose genetically inferred sex was discordant with self-reported sex assigned at birth, or assigned as other or intersex, to mitigate potential sample errors. We used cohort-provided genetic kinship coefficients to exclude one individual in each pair of individuals with first-degree or closer relatedness (kinship coefficient > 0.1768), preferentially removing controls in case-control pairs.^29^^,^^35^^,^^36^ Kinship coefficients were estimated using KING and PC-Relate for the UKB and AoU, respectively.^36^^,^^37^ ICD code definitions and case counts after filtering for each cancer type by cohort are presented in Table S1.

### Cancer group definitions and case-control sets

For our exome-wide pleiotropy screen, we defined three mutually exclusive biological cancer groupings based on the germ layer of origin (ectoderm, mesoderm, and endoderm).^19^^,^^38^^,^^39^^,^^40^ Cancer sites with highly heterogeneous germ layer origins were not assigned to a biologic group. We also defined three non-mutually exclusive etiological cancer groupings (hormone-related, smoking-related, and infection-related), assigning cancers to groups when risk factors were supported by three or more published reports.^20^^,^^21^^,^^22^^,^^23^^,^^24^^,^^25^^,^^26^^,^^27^^,^^28^ These cancer groupings (Table S2) were intended to represent selected, rather than all possible, biological and etiological classifications to identify pleiotropic associations. In total, we formed six case-control sets comprising cancer group individuals and a common pool of cancer-free control subjects within each cohort (Table S3). For analyses of individual cancer types, we created cancer-specific case-control sets using the same control subject pool. Cancer-free control subjects were defined as individuals with no cancer registry/EHR ICD-9 or ICD-10 codes or self-reported prior cancer diagnosis. We selected individual cancers with at least 500 affected individuals in at least one of the cohorts (Tables S4 and S5), including rare cancers defined as those with age-adjusted incidence rates < 15 affected individuals per 100,000 people in US 2018–2022 estimates from Surveillance, Epidemiology, and End Results (SEER) data (Table S6).^41^

### Genetic data collection and processing

In the UKB, blood samples were collected at enrollment and used for genotyping and genome sequencing. We analyzed genotyping and whole-exome sequencing (WES) data generated and pre-processed as described previously.^11^^,^^29^^,^^42^ The UKB WES dataset of 469,589 participants contained approximately 27 million genetic variants in coding regions. In the AoU, blood or saliva samples were collected at the time of consent and used for genotyping and whole-genome sequencing (WGS). We analyzed the AoU Controlled Tier Dataset v8 genotyping and short-read WGS (srWGS) data generated and pre-processed as described previously.^35^ The AoU srWGS dataset was pre-filtered by AoU to 45.7 million genetic variants in exonic regions within Gencode v.42 basic transcripts. We applied additional variant exclusions based on cohort-specific quality control (QC) criteria (supplemental methods).^43^^,^^44^

We used Ensembl Variant Effect Predictor (VEP) release 112 with the Combined Annotation Dependent Depletion (CADD) v.1.7, REVEL, and AlphaMissense plugins to annotate variants in the WES and srWGS datasets.^45^^,^^46^^,^^47^^,^^48^ We restricted annotations to variants in exons of MANE Select transcripts.^49^ Variants were assigned as predicted loss of function (pLoF) if they had one of the following VEP consequences: transcript ablation, splice acceptor variant, splice donor variant, stop gained, frameshift variant, stop lost, start lost, transcript amplification, feature elongation, and feature truncation.^45^ Variants were assigned as deleterious missense (missense) variants if they met one of the following criteria: (1) VEP missense consequence and a Phred-scaled CADD^46^ score > 20 or (2) VEP missense consequence with a REVEL^47^ score > 0.5, ClinVar^50^ or AlphaMissense^48^ annotation of “pathogenic” or “likely pathogenic,” a SIFT^51^ score annotation of “deleterious” or “deleterious low confidence,” or a PolyPhen2^52^ score annotation of “probably damaging” or “possibly damaging.” Synonymous variants were assigned to a synonymous variant set for each gene. Variants with other VEP consequences were included in a separate gene-based set if they had Phred-scaled CADD scores > 20. We constructed six variant sets based on combinations of the four variant annotation categories. The final annotation-based cRV sets were (1) pLoF, (2) missense, (3) pLoF and missense, (4) synonymous, (5) missense and synonymous, and (6) all cRVs. We also constructed separate annotation-based cRV sets restricted to pLoF and missense cRVs with a ClinVar classification of pathogenic or likely pathogenic (P/LP) as annotated by VEP.^45^ To define these, we searched the ClinVar^50^ annotations for pLoF and missense variants with terms “pathogenic,” “likely_pathogenic,” or “pathogenic/likely_pathogenic” and without the term “conflicting_interpretations_of_pathogenicity.” All cRVs with frequencies between a minor-allele count (MAC) of 11 and a minor-allele frequency (MAF) of 0.01 were also tested individually using single-variant tests.

In the UKB, we extracted the top 20 principal components (PCs) calculated by the UKB. We constructed a sparse genetic relationship matrix (GRM; GRM relatedness cutoff = 0.05) calculated from 236,413 high-quality, linkage-disequilibrium (LD)-pruned (r^2^ < 0.02) common variants in the UKB genotyping dataset using PLINK 2.0 (alpha v.6.5) and SAIGE (v.1.4.3).^53^^,^^54^^,^^55^ We calculated the top 30 PCs in AoU from 155,536 high-quality, LD-pruned (r^2^ < 0.01) common variants (MAF ≥ 0.01) in the AoU Global Diversity Array (GDA) genotyping dataset using PLINK 2.0.^53^^,^^54^ We constructed a sparse GRM (GRM relatedness cutoff = 0.175) calculated from the same set of common variants using SAIGE.^55^ A larger kinship coefficient threshold was used for the AoU sparse GRM due to the increased number of close genetic relationships (e.g., 4^th^ degree or closer) observed in that cohort. Variants in known regions of long-range high LD were excluded from PC and GRM calculations.^56^^,^^57^

### Statistical analyses

We tested cRV variant sets using generalized linear mixed models (GLMMs) implemented in SAIGE/SAIGE-GENE+ to account for population structure and relatedness.^55^^,^^58^^,^^59^ Each GLMM included adjustment for age at DNA sample collection, genetic sex, and PCs (20 for the UKB and 30 for AoU), with random effects modeled from cohort-specific GRMs. The number of PCs used in each cohort was selected based on the number used for previous RV analyses in these cohorts and by examining pairwise PC plots for study participants. A larger number of PCs were selected for AoU models due to the more complex population structure present in that cohort. We conducted single-variant analyses for individual cRVs with MAC > 10 and MAF < 0.01 using SAIGE. For gene-based analyses, we conducted MAF-weighted sum (WS) burden tests for associations between cancer groups and six annotation-based cRV sets using SAIGE-GENE+.^58^^,^^59^ Synonymous cRV sets were included to assess model robustness, as we expect the majority of these sets to have null associations. Cohort-specific estimates were meta-analyzed using fixed-effect methods implemented in the *metafor* R package (v.4.8-0) in R (v.4.3.2).^60^^,^^61^

To identify pleiotropic genes, we initially selected any genes that showed significant associations with at least one cancer group, based on either (1) a gene-based WS burden test in either cohort-specific or meta-analysis results or (2) a single cRV test in the meta-analysis and at least one cohort-specific analysis. We then evaluated these genes in 27 individual cancer types using cohort-specific SAIGE and SAIGE-GENE+ analyses.^55^^,^^58^^,^^59^ For 19 cancers, analyses included the full set of control subjects and the same GLMM models as described above for the cancer group analyses. For sex-specific cancers (breast, prostate, ovarian, endometrial, cervix, and testis), we restricted analyses to individuals of the genetic sex that the cancer most commonly occurs in and did not adjust for sex. We were not able to fit a full-sample GLMM for eye and non-melanoma skin cancers (NMSCs) in AoU, and we therefore applied a more stringent relatedness pruning threshold (kinship coefficient > 0.1) and randomly selected 25% of available controls for those two analyses. For single-cRV cancer group analyses, we used a standard significance threshold of *p <* 5 × 10^−8^. We used Bonferroni-corrected significance thresholds for single-cRV tests in individual cancer analyses (*p <* 0.05/number of variants) and all gene-based WS burden tests (*p <* 0.05/number of genes tested × 6 annotation-based sets per gene), recognizing that the latter is conservative given there are overlaps in annotations.

To obtain more interpretable risk effect estimates, we calculated cancer-specific odds ratios (ORs) associated with the presence of at least one cRV in the pLoF or missense variant sets for each potential pleiotropic gene. We performed these “carrier” status analyses using dichotomous (DC) burden tests with Firth’s penalized logistic regression, adjusting for the same fixed effects covariates as in the SAIGE models. We also repeated these tests restricting the analyses to ClinVar-annotated P/LP variants only. We fit penalized logistic regression models using the *brglm* R package (v.0.7.3)^62^ and meta-analyzed cohort-specific analyses as described above for the WS burden tests. As these DC burden tests lacked adjustment for random effects from sparse GRMs, they were only interpreted in cases where an association had been detected by the more robust WS burden tests.

## Results

### Participant and variant characteristics

After sample filtering, the number of affected individuals per cancer group ranged from 30,221 to 107,092 (Table S3), with 565,852 cancer-free control subjects available for analyses. Cancer-specific affected individual counts ranged from 808 (eye cancer) to 51,152 (NMSC) (Tables S4 and S5). Of the 27 individual cancer types tested, 16 (anus, bone, brain, esophagus, eye, Hodgkin lymphoma, leukemias, liver, myeloma, neck, oral, ovary, pancreas, stomach, testis, and thyroid) met the definition of a rare cancer.

After variant annotation and QC filtering, we included 7,391,304 cRVs across 17,467 genes (UKB) and 8,768,765 cRVs across 17,801 genes (AoU) in gene-based WS burden tests (Table S7). In the UKB, 9%, 50%, 39%, and 3% cRVs were annotated as pLoF, missense, synonymous, and other, respectively, with corresponding numbers for AoU being 10%, 47%, 38%, and 5%. We assessed up to 17,389 genes in both cohorts. The number of variants analyzed in single-cRV tests ranged from 1,083,488 (14.7% of total variants included in gene-based tests) to 1,195,135 (16.2%) in the UKB and 1,164,814 (13.3%) to 1,228,029 (14.0%) in AoU, depending on the cancer group (Table S7). The number of individual cRVs eligible for meta-analysis ranged from 654,719 to 710,233 across cancer groups.

### Exome-wide pleiotropy cRV screening tests

**Figure 1:**
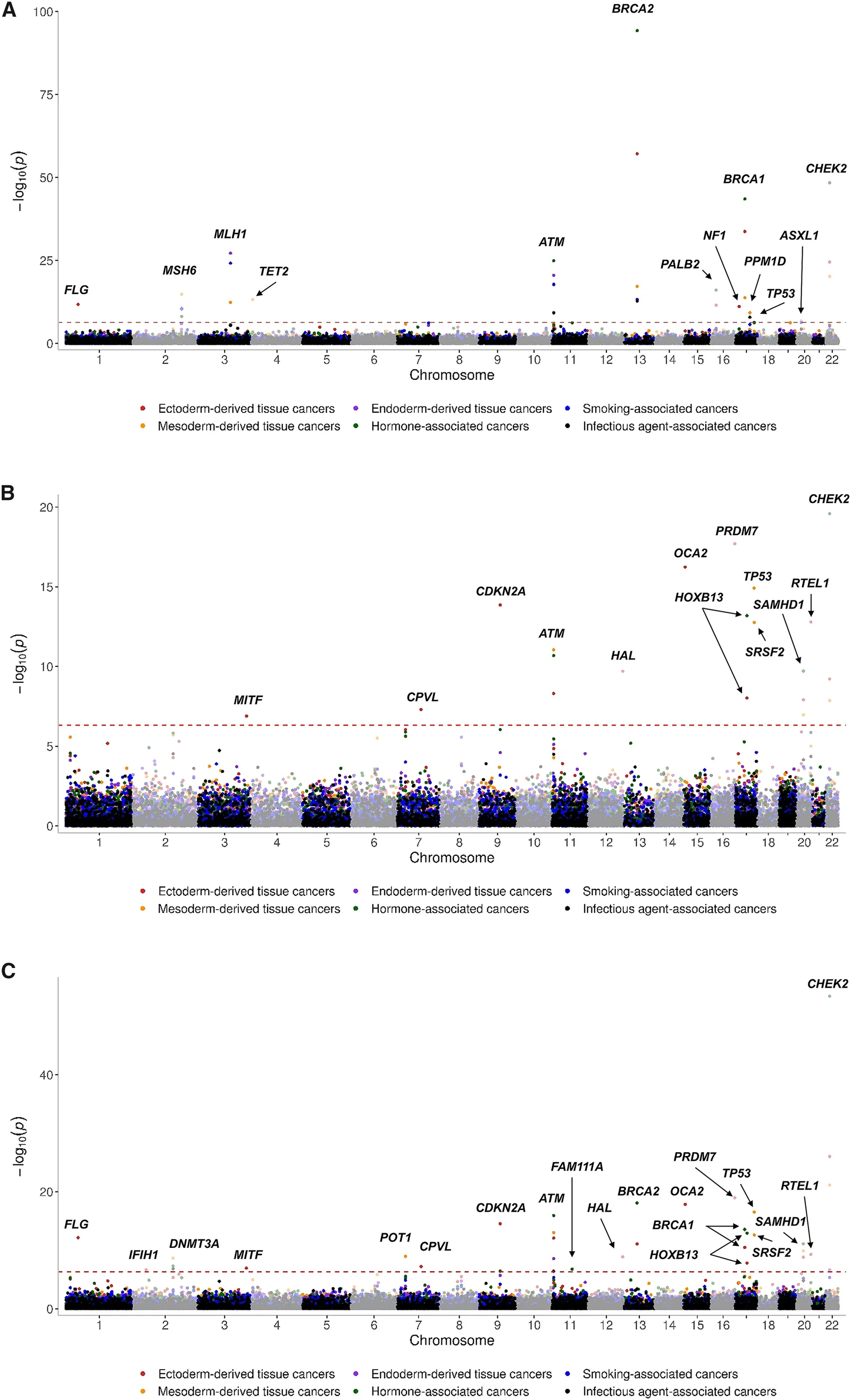
Manhattan plots showing gene-based MAF-weighted sum burden test meta-analysis results for the exome-wide pleiotropy screening tests in the six cancer groups (A) pLoF cRV sets. (B) Missense cRV sets. (C) pLoF and missense cRV sets. Results for all six cancer groups are included in each Manhattan plot in different colors (red: ectoderm-derived tissue cancers, orange: mesoderm-derived tissue cancers, purple: endoderm-derived tissue cancers, green: hormone-associated cancers, blue: smoking-associated cancers, and black: infectious agent-associated cancers). The red dashed line represents the Bonferroni-corrected significance threshold of *p =* 4.79 × 10^−7^ (0.05/17,389 genes × 6 cRV sets).

We observed no evidence for genome inflation in the exome-wide gene-based or single cRV tests (Figures S1–S22, supplemental methods). In the gene-based analyses, we identified 27 genes where at least one cRV set was significantly associated (*p <* 4.79 × 10^−7^ [0.05/17,389 genes × 6 cRV sets]) with at least one cancer group in the meta-analyses (Figure 1; Table S8). At least one of the cRV sets for *ATM* and *BRCA2* was significantly associated with all six cancer groups, with the strongest association observed between *BRCA2* pLoF variants and hormone-associated cancers (Table S8). *ATM* showed consistent associations across pLoF and missense cRV sets for all six cancer groups. Of the 27 genes identified in the meta-analyses, 25 were significant in the UKB, and 13 were significant in AoU (Table S8). Three additional genes (*PLEKHA4*, *HLA-DPB1*, and *MICA*) were significant in the UKB only. We also identified three genes (*JAK2*, *IDH2*, and *IGLL5*) where single cRVs were significantly associated (*p <* 5 × 10^−8^) with at least one cancer group in the meta-analyses and one of the cohorts (Table S9). Overall, we identified 33 potentially pleiotropic genes that were further analyzed in the individual cancer analyses.

### Individual cancer cRV tests

**Figure 2:**
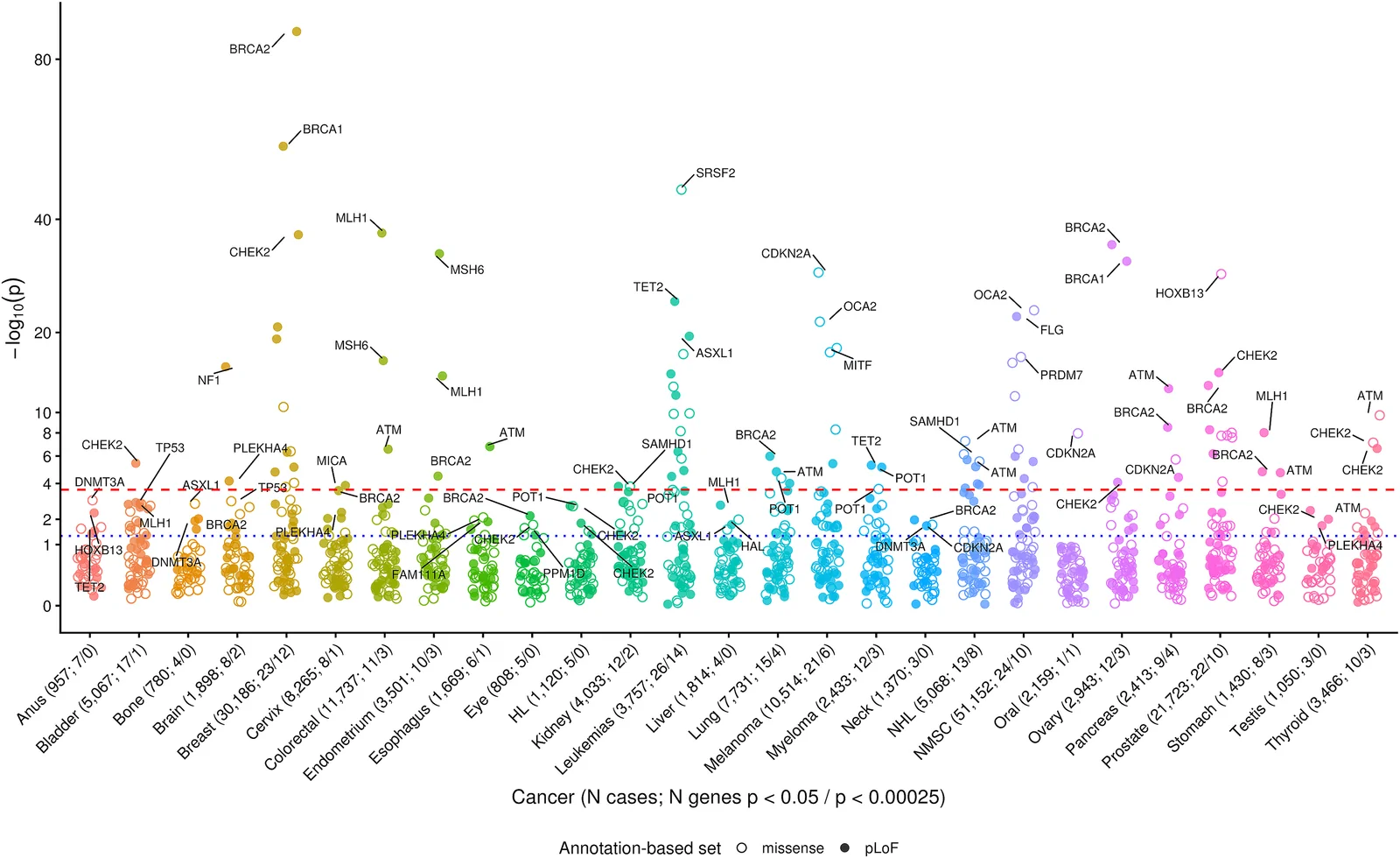
Plot showing gene-based MAF-weighted sum burden test meta-analysis results for pLoF and missense variant sets in 33 genes across 27 individual cancers Results are grouped and colored by cancer type. Missense variant sets are represented by filled circles, and pLoF variant sets are represented by empty circles. The top three associations with *p <* 0.05 for each cancer are labeled. The red dashed line represents the Bonferroni-corrected significance threshold of *p =* 0.00025 (0.05/33 tested genes × 6 cRV sets). The dashed blue line represents a nominal significance threshold of *p =* 0.05. The case counts and the number of genes associated at *p <* 0.05 and *p <* 0.00025 are indicated on the *x* axis.

**Figure 3:**
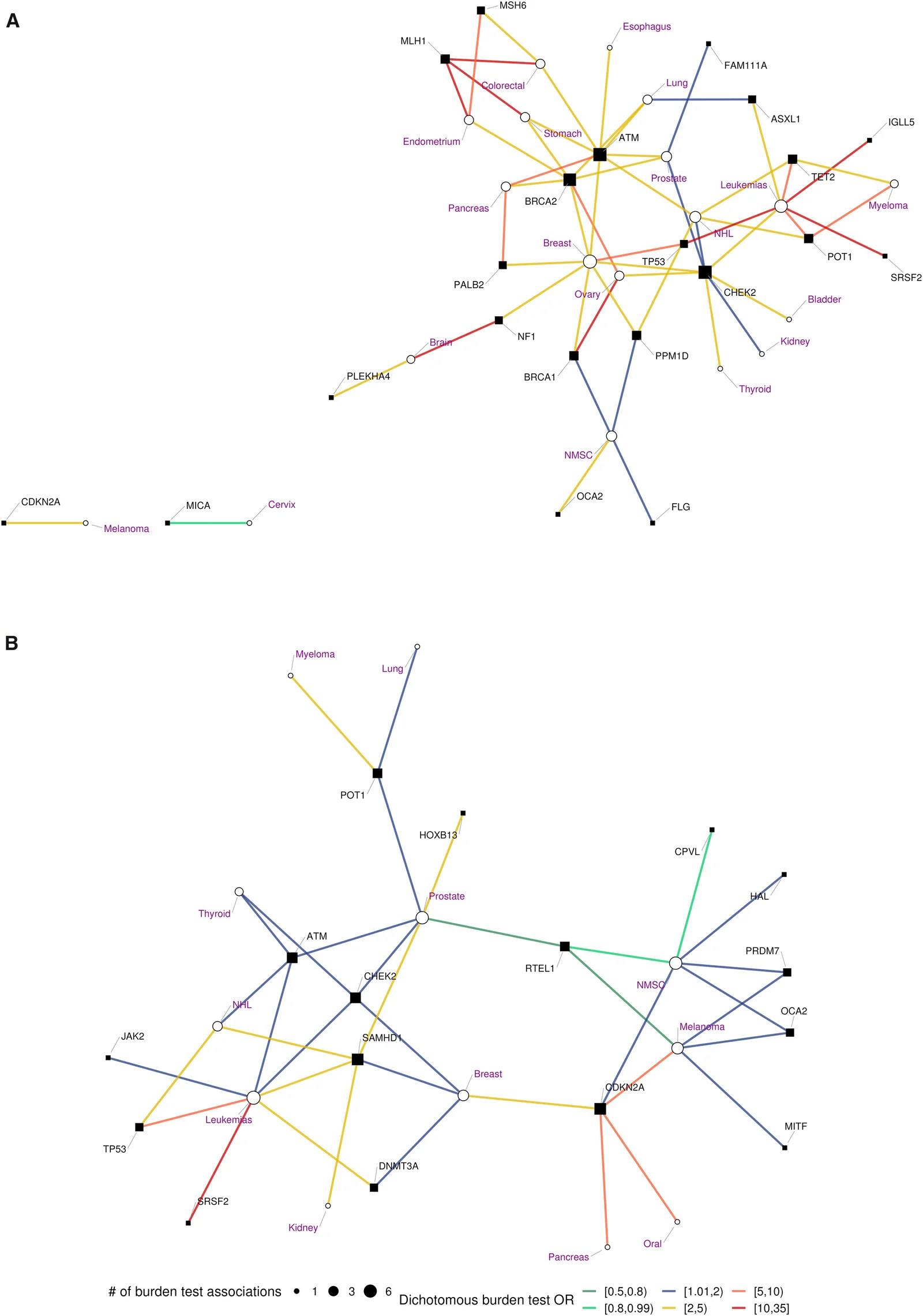
Network diagram showing significant relationships between gene-based sets of pLoF and missense variants and individual cancers Only associations from meta-analyses of MAF-weighted sum burden tests that were significant after Bonferroni correction for the number of burden tests performed (*p <* 0.00025 [0.05/33 tested genes × 6 annotation-based sets]) are plotted. Empty circles and purple text represent cancers. Filled squares and black text represent genes. Node sizes are proportional to the number of associations for each gene or cancer (node degree centrality). Edge colors represent odds ratio estimates for each association between gene-based cRV tests and cancers from dichotomous burden tests. NHL, non-Hodgkin lymphoma; NMSC, non-melanoma skin cancer. (A) pLoF variants. (B) Missense variants.

**Figure 4:**
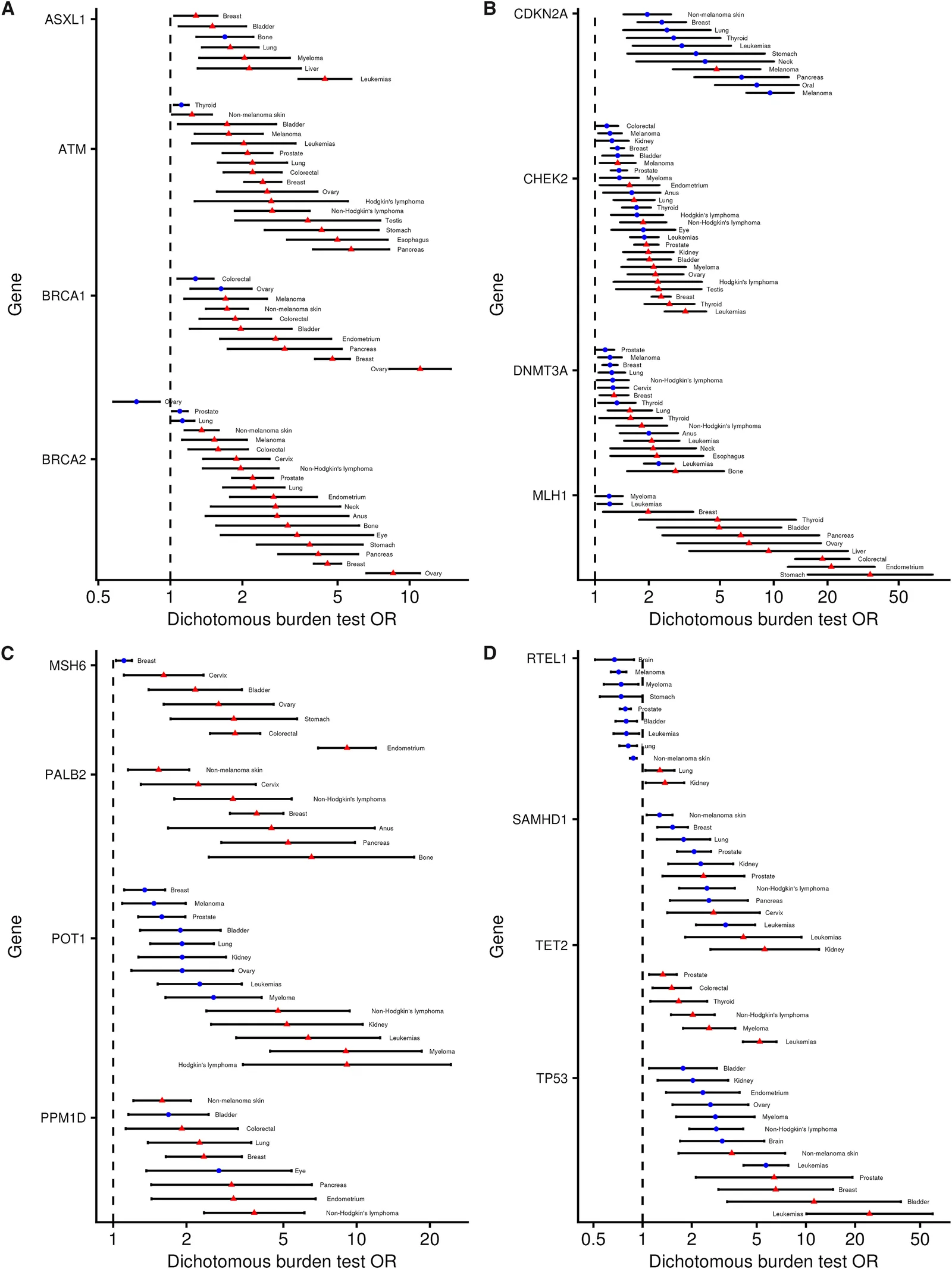
Forest plots of dichotomous burden tests with nominally significant associations in 16 broadly pleiotropic genes Each point represents an OR estimate from a meta-analyzed dichotomous burden test that was nominally significant (*p <* 0.05 in both burden test types) for a cancer site as indicated by the text label. OR estimates are grouped by gene, with red triangles and blue circles representing associations from pLoF and missense variant sets, respectively, and plotted on a log base 10 *x* axis scale. Black error bars represent 95% confidence intervals. The dashed vertical bar represents OR = 1. Dichotomous burden test ORs for (A) *ASXL1*, *ATM*, *BRCA1*, and *BRCA2*; (B) *CDKN2A*, *CHEK2*, *DNMT3A*, and *MLH1*; (C) *MSH6*, *PALB2*, *POT1*, and *PPM1D*; and (D) *RTEL1*, *SAMHD1*, *TET2*, and *TP53*.

In gene-based burden tests across 33 genes and 27 cancer types, we identified 91 significant cancer-gene associations (*p* < 0.00025 [0.05/33 genes × 6 cRV sets]), of which 89 involved pLoF, missense, or pLoF + missense cRV sets (Figures 2 and 3; Table S10). The remaining two associations were for anus cancer and *MITF* (three annotation sets: all, missense + synonymous, and synonymous variants) and for melanoma and *PPM1D* (synonymous variants). For 15 genes (*ASXL1*, *ATM*, *BRCA1*, *BRCA2*, *CDKN2A*, *CHEK2*, *DNMT3A*, *MLH1*, *MSH6*, *PALB2*, *POT1*, *PPM1D*, *SAMHD1*, *TET2*, and *TP53*), the gene-based burden tests for pLoF and missense cRV sets showed consistent ORs > 1 across cancer types. In contrast, missense cRVs in *RTEL1* showed consistent protective associations (ORs < 1) across cancer types, including brain (OR_DC_burden_ = 0.67, 95% confidence interval [CI]: 0.51–0.89), myeloma (OR_DC_burden_ = 0.71, 95% CI: 0.64–0.80), and prostate cancer (OR_DC_burden_ = 0.78, 95% CI: 0.72–0.85). For 17 genes (*CPVL*, *FAM111A*, *FLG*, *HAL*, *HLA-DPB1*, *HOXB13*, *IDH2*, *IFIH1*, *IGLL5*, *JAK2*, *MICA*, *MITF*, *NF1*, *OCA2*, *PLEKHA4*, *PRDM7*, and *SRSF2*), OR estimates were less consistent across cancers, with significant associations observed only for a subset of cancer types (Figures 3 and 4; Table S10). In general, pLoF variants had larger ORs than missense variants.

The OR estimates from WS and DC burden tests were highly concordant (Spearman rank ⍴ = 0.89 for pLoF and 0.88 for missense sets; Figure S23), with consistent OR patterns observed across genes and cancer types (Figures S24 and S25). The DC burden test OR estimates for significant gene-cancer pairs tended to be larger but less precise than WS burden test estimates. There was also substantial concordance in ORs based on the full cRV sets and ORs based on ClinVar-filtered cRV sets (Figure S26).

Single-variant tests of 1,468 single cRVs in the 33 genes (Table S11) identified 49 significant associations (*p <* 3.4 × 10^−5^ [0.05/1,468]), representing 32 unique pLoF and missense cRVs. The majority of these associations were observed in breast cancer (*n* = 10), leukemias (*n* = 10), melanoma (*n* = 7), and prostate cancer (*n* = 5). Several ClinVar pLoF variants showed robust multi-cancer associations. For example, the *CHEK2* frameshift variant rs555607708 was associated with cancers of the breast (OR = 2.6; 95% CI 2.2–3.0; *p* = 9.2 × 10^−33^), kidney (OR = 2.6; 95% CI 1.7–4.0; *p* = 2.2 × 10^−5^), prostate (OR = 1.99; 95% CI 1.63–2.44; *p* = 2.6 × 10^−11^), thyroid (OR = 2.86; 95% CI 1.85–4.42; *p* = 2.1 × 10^−6^), and leukemias (OR 3.4; 95% CI 2.36–4.91; *p* = 6.3 × 10^−11^). In the UKB, 132 individuals with cancer (0.13%), compared to 319 control subjects (0.10%), had more than one pLoF cRV across the 33 genes. The corresponding number in AoU was 210 affected individuals (0.43%) and 482 control subjects (0.19%) (Tables S12 and S13).

## Discussion

Using data from two large population-based biobanks, we identified 33 genes that show pleiotropic cRV associations with cancer. Not surprisingly, the majority of significant gene associations were observed for the sets of pLoF or missense cRVs. We observed wide-spread pleiotropy for the well-known cancer predisposition genes *ATM*, *BRCA2*, and *CHEK2*, with significant associations (*p* < 2.5 × 10^−4^) for 16 of the 27 cancer types tested, including breast, ovary, and prostate (all three genes); lung, non-melanoma, pancreas, and stomach (*ATM* and *BRCA2*); bladder, Hodgkin lymphoma, leukemias, and non-Hodgkin lymphoma (*ATM* and *CHEK2*); endometrium (*BRCA2* only); colorectal, esophagus (*ATM* only); and kidney and thyroid (*CHEK2* only). These three genes have established roles in cancer-related biological processes and are implicated in hereditary cancer syndromes that increase risk for multiple cancers.^63^ Our results suggest that these genes may play important roles in carcinogenesis across an even broader range of cancer types, including rare cancers (Figures 3 and 4). As anticipated, we observed an extremely limited number of exome-wide significant associations for synonymous-variant-only cRV sets, and the lack of inflation of these test statistics provided robust evidence that our findings were not driven by systematic bias.

All 33 of the potential pleiotropic genes we identified have previously been linked to at least one cancer,^64^^,^^65^^,^^66^^,^^67^^,^^68^^,^^69^^,^^70^^,^^71^^,^^72^^,^^73^^,^^74^^,^^75^^,^^76^^,^^77^ with previous associations often observed for breast, leukemias, non-melanoma skin, melanoma, and prostate. This is not surprising given the comparatively large case counts for most of these cancers and the distinct genetic etiology of leukemias reported in previous studies.^78^

We observed that missense cRVs in *RTEL1* were nominally associated with decreased odds of cancer diagnosis across a range of cancer types, including bladder, brain, leukemia, lung, melanoma, myeloma, non-melanoma, prostate, and stomach. *RTEL1* encodes a DNA helicase involved in maintaining telomere stability.^79^ Telomere length is associated with cancer risk, and a recent Mendelian randomization analysis observed associations between longer telomere length and increased risk for breast, colorectal, kidney, lung, ovarian, and prostate cancer.^80^ cRVs in *RTEL1* have shown complex directional allelic heterogeneity in telomere length associations, with different variants having opposing effects,^81^ supported by molecular studies showing that both increased and decreased RTEL1 expression may be involved in genomic instability related to RTEL1’s role in unwinding DNA G-quadruplexes and maintaining replication fork progression, contributing to multiple human conditions, including malignancies.^79^ Increased RTEL1 expression has been observed in colorectal and stomach tumors, and cancer GWASs have identified common variant associations between *RTEL1* and colorectal, kidney, and prostate cancer.^64^^,^^82^^,^^83^ Our results corroborate this previous work and suggest that cRVs in *RTEL1* may be involved in the etiology of more cancers than previously reported, warranting further investigation.

Effect sizes for pLoF and missense cRVs varied across cancer types, but magnitudes were generally consistent with prior studies. For example, pLoF results for *ATM*, *BRCA2*, *CHEK2*, and *PALB2* were consistent with previous studies of germline pathogenic RVs in a number of cancers.^84^^,^^85^^,^^86^^,^^87^^,^^88^^,^^89^ All significant single cRVs had been reported in ClinVar,^50^ but identifying significant associations in a population-based study of germline genetic variation and cancer provides additional support for their impact at the population level.

Eleven genes (*ASXL1*, *ATM*, *CHEK2*, *DNMT3A*, *IGLL5*, *JAK2*, *POT1*, *SAMHD1*, *SRSF2*, *TET2*, and *TP53*) were significantly associated with increased odds of diagnosis with leukemia. As a large proportion of these genes are frequently mutated in individuals with clonal hematopoiesis of indeterminate potential (CHIP), caution is warranted in interpreting these associations. Our analyses included prevalent cases of leukemia, and there is evidence that a relatively high proportion (40%–50%) of LoF mutations in CHIP-associated genes in UKB WES data may represent somatic mutations.^42^ However, we found little evidence that prevalent case status was associated with increased cRV burden in our analyses (Figure S27; supplemental methods). CHIP-associated genes such as *ASXL1* and *DNMT3A* are strongly linked to hematopoietic cancers,^90^^,^^91^ but we also observed associations for these genes with lung and breast cancer in accordance with previous studies of somatic mutations in these cancer types.^92^^,^^93^^,^^94^

Strengths of this study include the large sample size (*n* = 729,469), enabling analyses of gene-based RV associations for rare cancers that remain understudied despite making up a sizable proportion of the cancers diagnosed annually.^1^^,^^41^ Further, we did not exclude samples due to inferred genetic ancestry, enabling a more diverse set of cRVs and enhancing generalizability of the study findings. Most prior analyses of cRVs and cancer risk were conducted in populations with genetic ancestry similar to European reference populations.^11^^,^^12^^,^^13^^,^^14^^,^^15^^,^^16^^,^^17^^,^^95^ The inclusion of all eligible UKB or AoU participants regardless of inferred genetic ancestry enabled the inclusion of cRVs that may be specific to understudied populations.^96^ Compared to common variant associations, gene-based RV associations tend to be consistent across genetic ancestry populations in studies of non-cancer traits.^97^^,^^98^ However, it is important to note that the majority of participants (95% of UKB participants and 49% of AoU participants) in this study have genetic ancestry similar to European reference populations,^35^^,^^99^ and thus our analyses have a large European-centric bias.

Our study also had some limitations. We limited our analyses to coding regions, even though recent evidence suggests that non-cRVs may be as or more impactful on risk for a variety of human traits.^100^ Non-cRVs are more challenging to annotate and group, and there is some evidence that exome-based sequencing datasets are more efficient for identifying RV associations in large populations.^101^ The majority of analyzed cRVs had fewer than 10 alleles, and while we only included variants that had passed QC procedures implemented by the UKB and AoU,^11^^,^^35^^,^^43^ we cannot rule out the possibility that some of these variants are technical artifacts. We also only utilized fixed-effect meta-analyses to maximize statistical power, but this approach may not have fully accounted for heterogeneity in RV effects across the two cohorts. Further, we did not assess relationships between family history of cancer and cRVs due to limited family history data. It is also important to note that ICD-9/10 codes may have increased the risk of case misclassification, and we did not have access to more refined molecular or histological subtyping that may be relevant for genetic studies (e.g., estrogen receptor status).^102^ In addition, we included both prevalent and incident cancer diagnoses to maximize statistical power, potentially introducing bias from somatic variant effects. Lastly, we chose to combine invasive and *in situ* cancer diagnoses to maximize statistical power, potentially missing associations exclusive to either one of them.

In summary, we identified 33 pleiotropic cancer genes with cRV associations for at least one cancer group. When examining associations between these 33 genes and 27 individual cancer types, results recapitulated many known relationships between genes and cancers but also implicated genes for a wider range of cancers. Examining groups of related cancers shows promise for characterizing genes where rare genetic variation may have pleiotropic effects across multiple cancer types. This may facilitate studies of rare cancers, where genetic epidemiological studies have lagged. Future studies with more refined cancer groupings could be used to elucidate the shared genetic architecture of closely related cancer types.

## Data and code availability

The individual-level genotype and phenotype data from the UKB are available through application to the UKB (http://www.ukbiobank.ac.uk). This study used data from the AoU Research Program’s Controlled Tier Dataset 8, available to authorized users on the Researcher Workbench. The code used in these analyses and additional summary statistics are publicly available in our project GitHub repository (https://github.com/UW-Epidemiology/multi_cancer_RVAS).

## Acknowledgments

This project was supported by 10.13039/100000054NCI grants U01 CA194393 (PI: S.L.), R03 CA287235 (PI: B.F.D.), P50 CA097186 (B.F.D.), and P30 CA015704 (B.F.D.). A.H.S. was supported by T32 CA009168 (NCI). The contents of this manuscript are solely the responsibility of the authors and do not necessarily represent the official views of the NCI, NIH. This project was also supported by Fred Hutchinson Cancer Center TDS IRC Pilot Award Grant 229305-99 (PI: B.F.D.). We are grateful to the UKB participants who have generously agreed to provide a broad range of information for health-related research. This research was conducted using the UKB Resource under application number 95770. We gratefully acknowledge AoU participants for their contributions, without whom this research would not have been possible. We also thank the NIH AoU Research Program for making available the participant data examined in this study.

## Author contributions

Conceptualization, A.H.S., S.L., B.F.D., and M.C.W.; data curation, A.H.S. and T.A.H.; formal analysis, A.H.S.; methodology, A.H.S., S.L., B.F.D., M.C.W., and J.Z.; visualization, A.H.S.; writing – original draft, A.H.S. and S.L.; writing – review & editing, B.F.D., M.C.W., T.A.H., and J.Z.; project administration, S.L., B.F.D., and T.A.H.; funding acquisition, S.L. and B.F.D.; supervision, S.L., B.F.D., and M.C.W.

## Declaration of interests

The authors declare no competing interests.

## Web resources

GitHub for multi-cancer RVAS summary statistics and analysis code (this study), https://github.com/UW-Epidemiology/multi_cancer_RVAS
UK Biobank, http://www.ukbiobank.ac.uk

## Supplemental information

Data S1. Tables S1–S13

Document S2. Article plus supplemental information
