# Supplementary material for: The pleiotropic landscape of rare variant associations with multiple cancers in large biobanks
Source: HGG Adv. 2026 Aug 20;7(4):100662. doi: 10.1016/j.xhgg.2026.100662 (PMC13571141; doi:10.1016/j.xhgg.2026.100662)
Supplement: Supplementary file 1 [file mmc1.pdf]

HGGA, Volume 7

Supplemental information

The pleiotropic landscape of rare variant associations with multiple cancers in large biobanks

Austin Hammermeister Suger, Tabitha A. Harrison, Jiachen Zhang, Michael C. Wu, Burcu F. Darst, and Sara Lindström

Table of Contents

Supplemental Figures 2

Supplemental Methods 33

Whole genome and whole exome sequencing variant quality control filtering criteria 33

Assessment of modeling assumptions in cancer group and single cancer analyses 33

Assessment of the influence of somatic mutations in clonal hematopoiesis of indeterminate potential (CHIP) genes 34

Supplemental References 34

Supplemental Figures

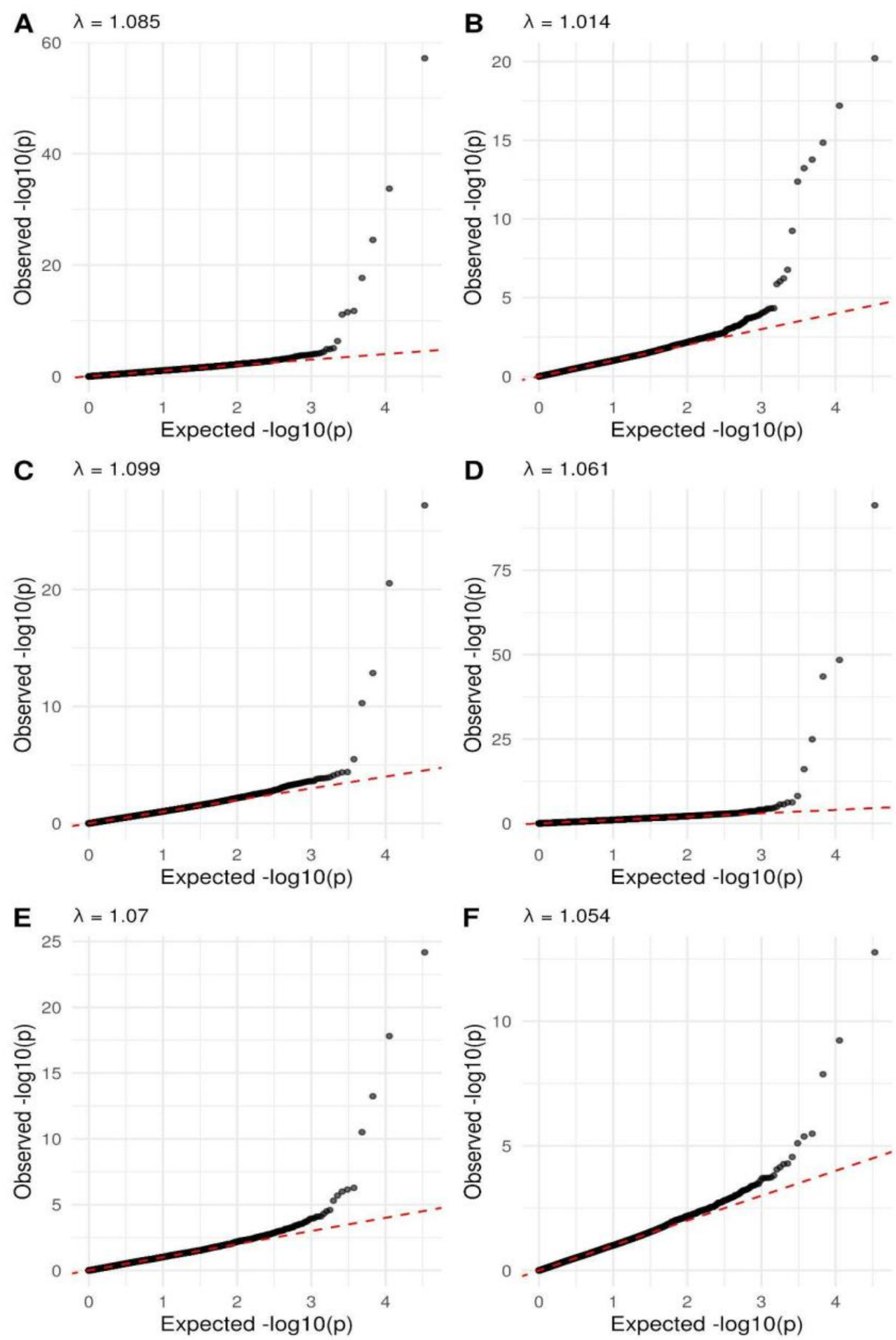

Figure S1. QQ plots with genomic inflation factors (λ) values for meta-analysis of SAIGE-GENE+ gene-based MAF weighted sum burden tests including pLoF variants only. A) ectoderm-derived tissue cancers, B) mesoderm-derived tissue cancers, C) endoderm-derived tissue cancers, D) hormone-associated cancers, E) smoking-associated cancers, F) infectious agent-associated cancers.

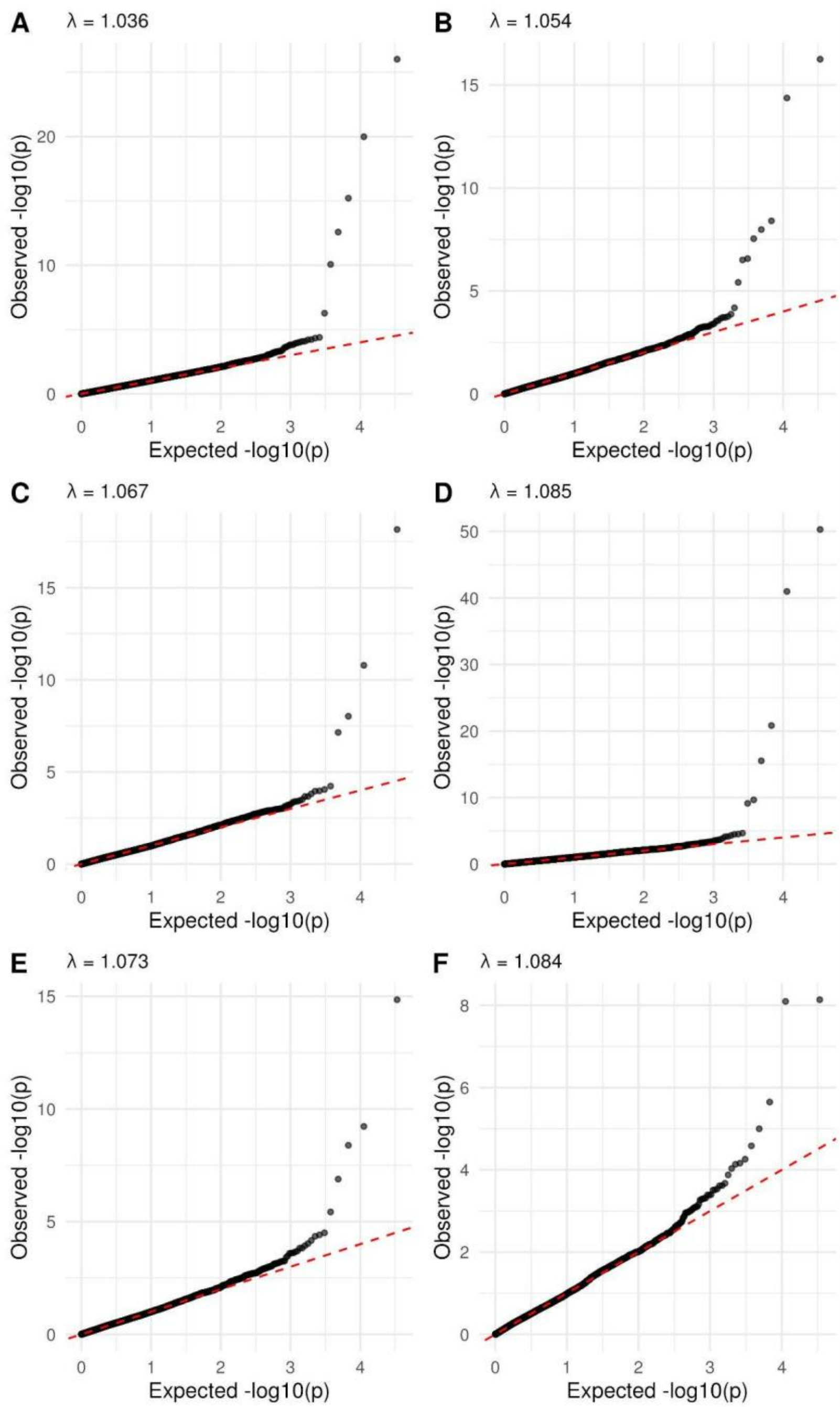

Figure S2. QQ plots with genomic inflation factors (λ) values for UKB SAIGE-GENE+ gene-based MAF weighted sum burden tests including pLoF variants only. A) ectoderm-derived tissue cancers, B) mesoderm-derived tissue cancers, C) endoderm-derived tissue cancers, D) hormone-associated cancers, E) smoking-associated cancers, F) infectious agent-associated cancers.

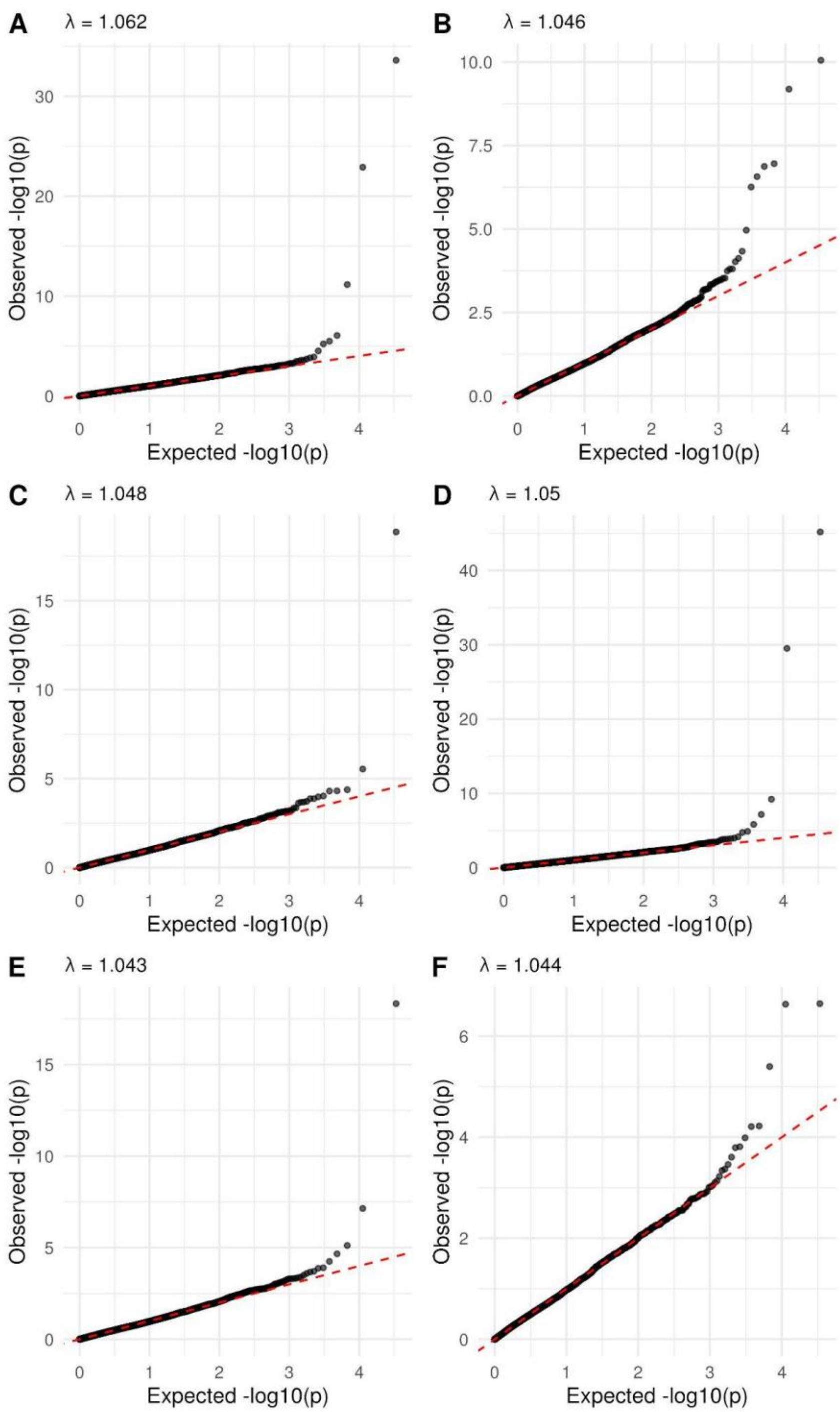

Figure S3. QQ plots with genomic inflation factors (λ) values for AoU SAIGE-GENE+ gene-based MAF weighted sum burden tests including pLoF variants only. A) ectoderm-derived tissue cancers, B) mesoderm-derived tissue cancers, C) endoderm-derived tissue cancers, D) hormone-associated cancers, E) smoking-associated cancers, F) infectious agent-associated cancers.

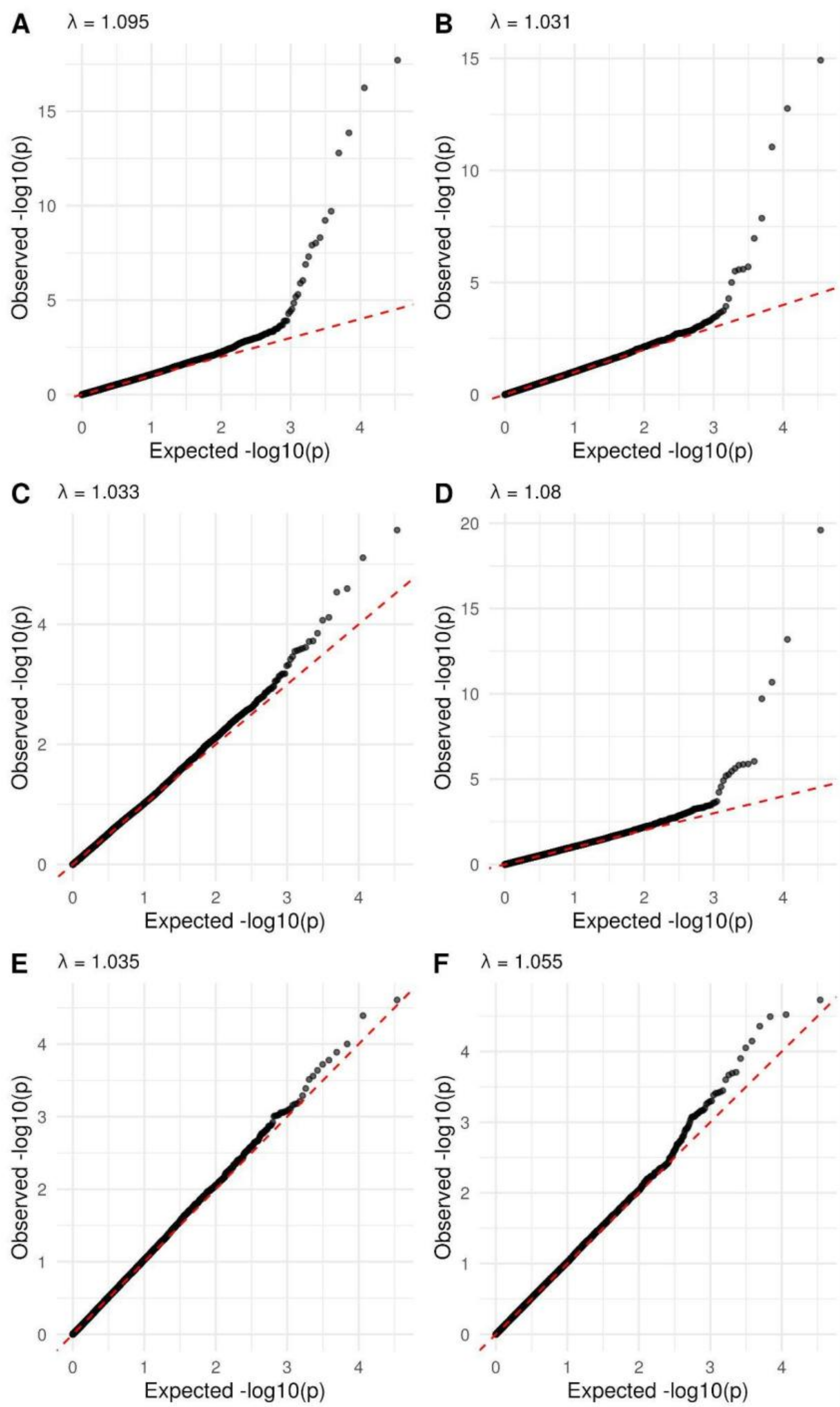

Figure S4. QQ plots with genomic inflation factors (λ) values for meta-analysis of SAIGE-GENE+ gene-based MAF weighted sum burden tests including missense variants only. A) ectoderm-derived tissue cancers, B) mesoderm-derived tissue cancers, C) endoderm-derived tissue cancers, D) hormone-associated cancers, E) smoking-associated cancers, F) infectious agent-associated cancers.

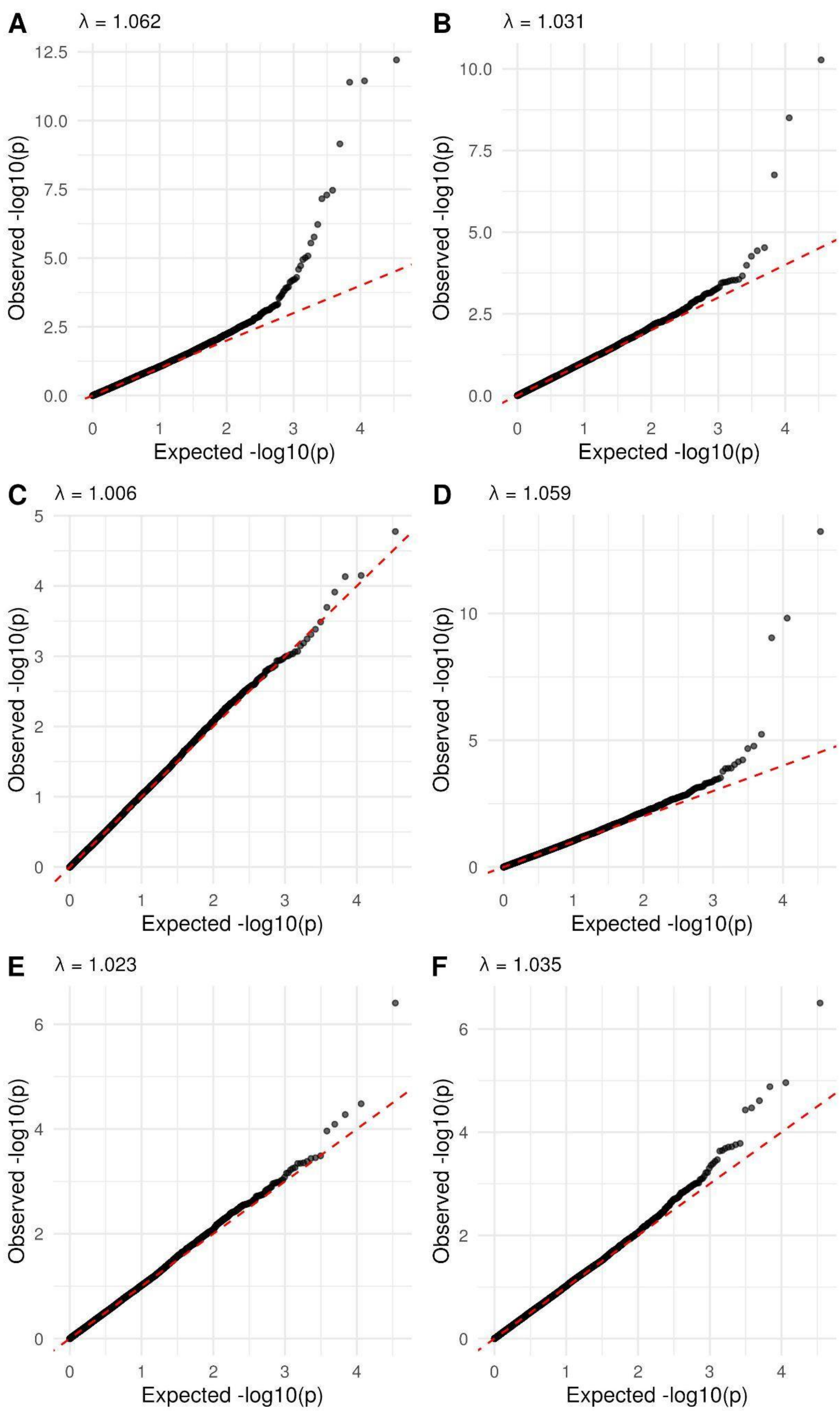
A
λ = 1.062
B
λ = 1.031
C
λ = 1.006
D
λ = 1.059
E
λ = 1.023
F
λ = 1.035
Observed -log10(p)
Expected -log10(p)

Figure S5. QQ plots with genomic inflation factors (λ) values for UKB SAIGE-GENE+ gene-based MAF weighted sum burden tests including missense variants only. A) ectoderm-derived tissue cancers, B) mesoderm-derived tissue cancers, C) endoderm-derived tissue cancers, D) hormone-associated cancers, E) smoking-associated cancers, F) infectious agent-associated cancers.

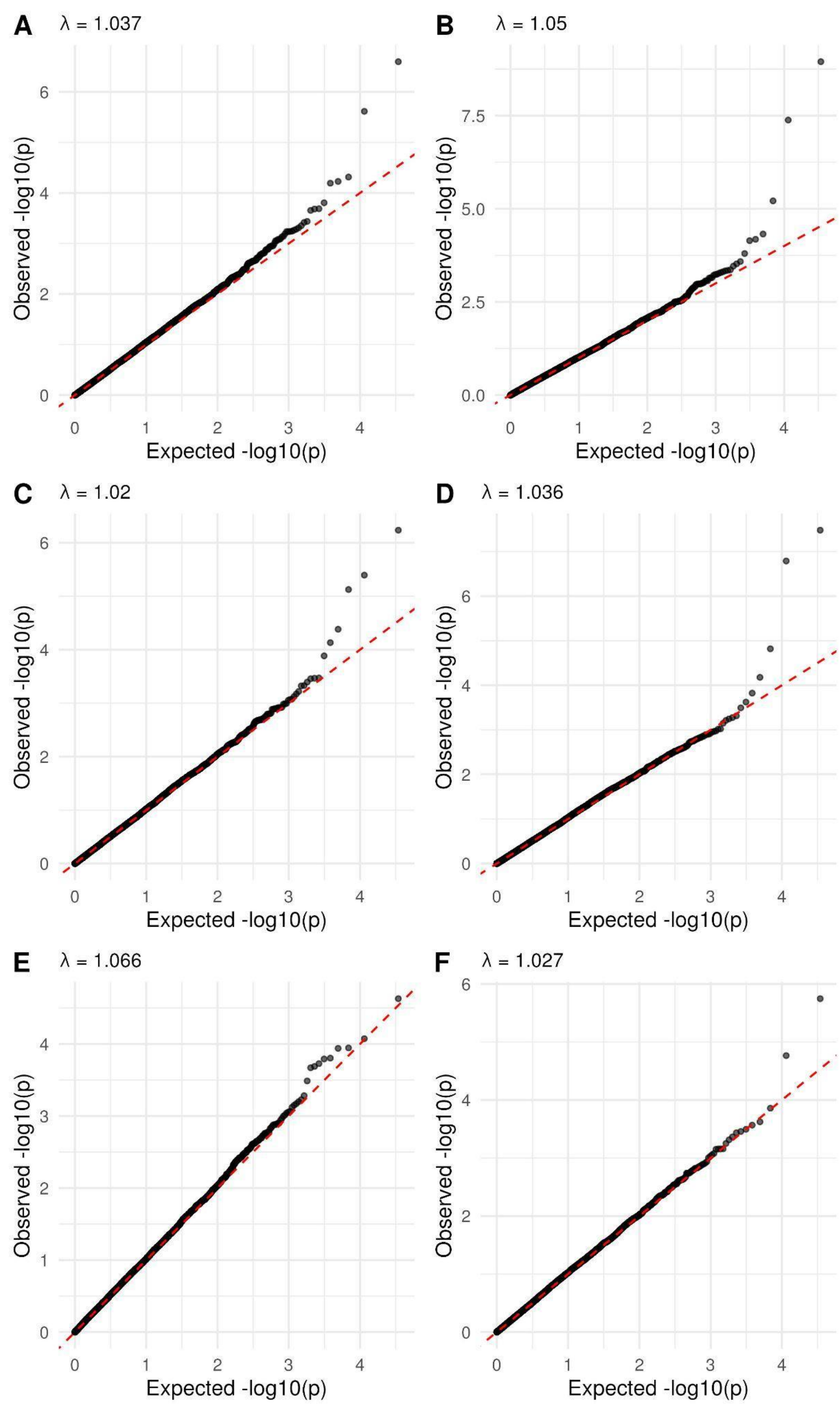

Figure S6. QQ plots with genomic inflation factors (λ) values for AoU SAIGE-GENE+ gene-based MAF weighted sum burden tests including missense variants only. A) ectoderm-derived tissue cancers, B) mesoderm-derived tissue cancers, C) endoderm-derived tissue cancers, D) hormone-associated cancers, E) smoking-associated cancers, F) infectious agent-associated cancers.

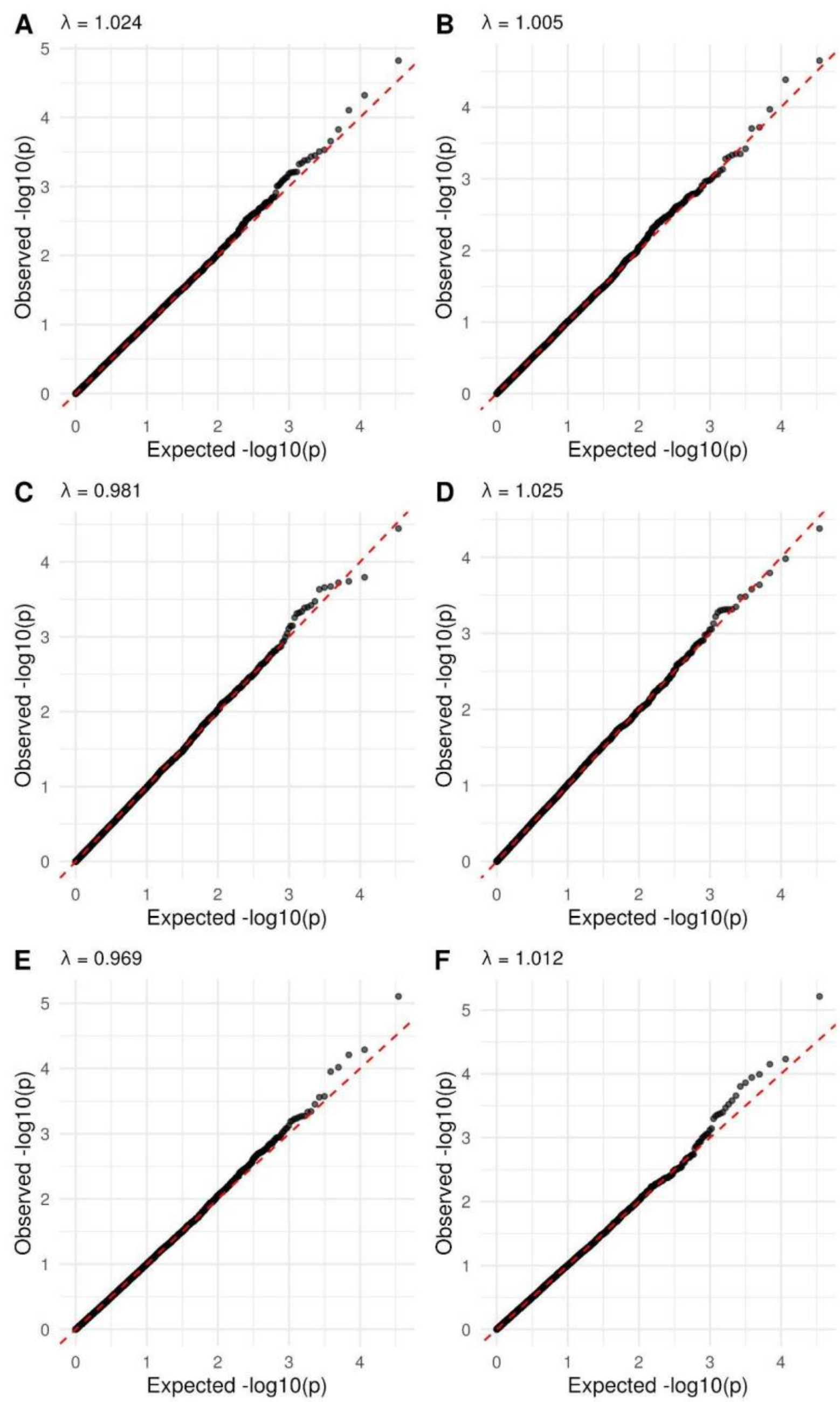

Figure S7. QQ plots with genomic inflation factors (λ) values for meta-analysis of SAIGE-GENE+ gene-based MAF weighted sum burden tests including synonymous variants only. A) ectoderm-derived tissue cancers, B) mesoderm-derived tissue cancers, C) endoderm-derived tissue cancers, D) hormone-associated cancers, E) smoking-associated cancers, F) infectious agent-associated cancers.

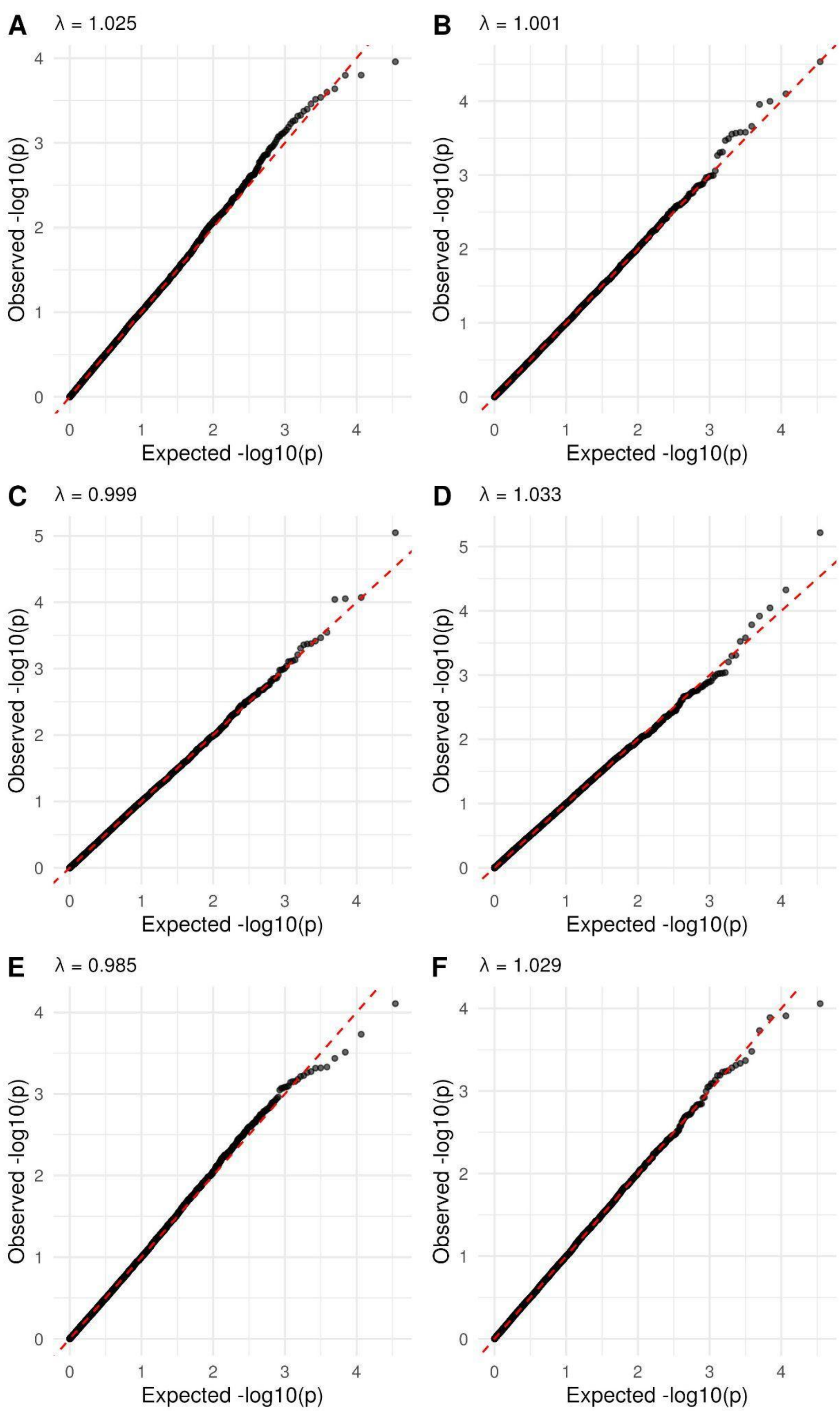
A
λ = 1.025
B
λ = 1.001
C
λ = 0.999
D
λ = 1.033
E
λ = 0.985
F
λ = 1.029
Observed -log10(p)
Expected -log10(p)

Figure S8. QQ plots with genomic inflation factors (λ) values for UKB SAIGE-GENE+gene-based MAF weighted sum burden tests including synonymous variants only. A) ectoderm-derived tissue cancers, B) mesoderm-derived tissue cancers, C) endoderm-derived tissue cancers, D) hormone-associated cancers, E) smoking-associated cancers, F) infectious agent-associated cancers.

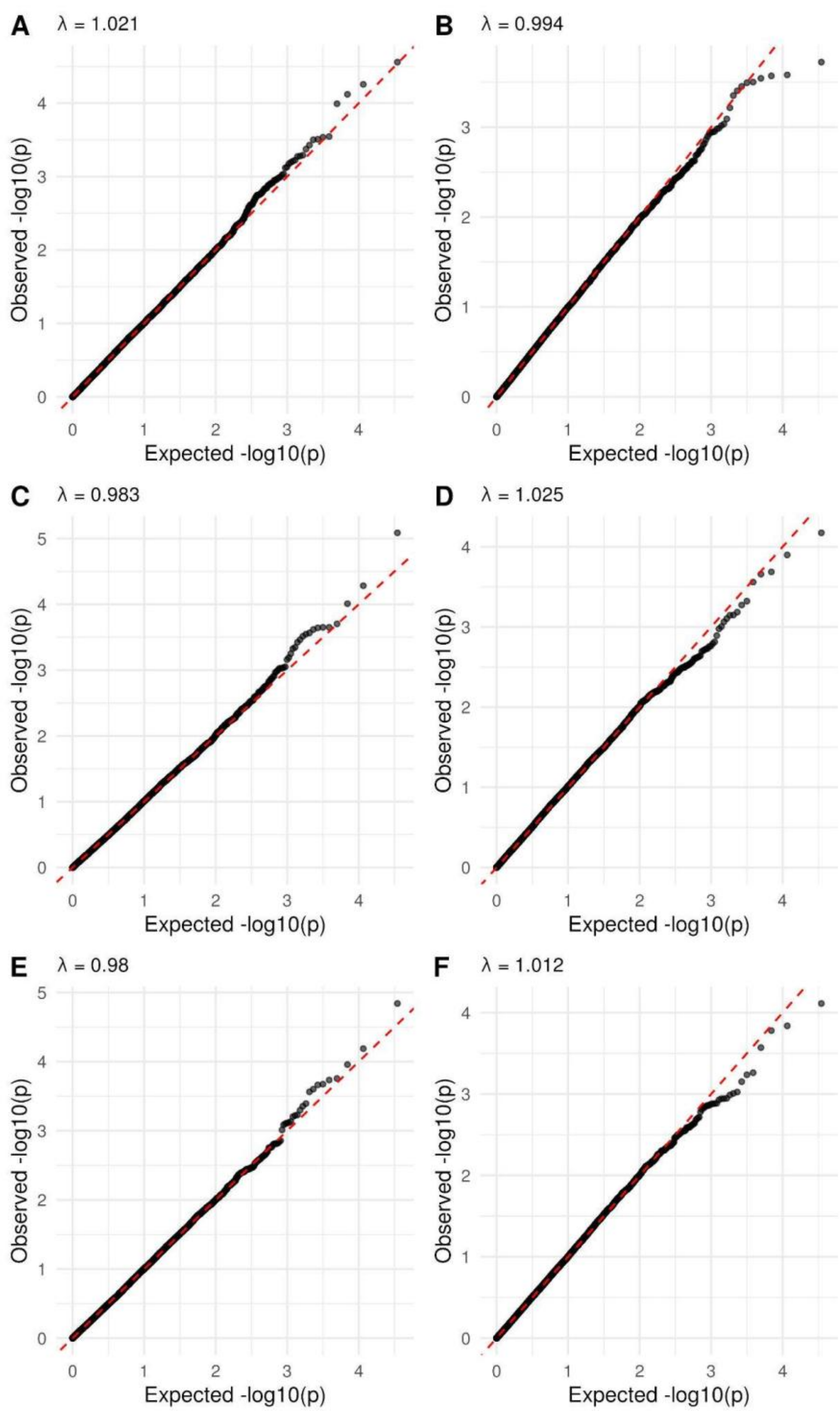

Figure S9. QQ plots with genomic inflation factors (λ) values for AoU SAIGE-GENE+ gene-based MAF weighted sum burden tests including synonymous variants only. A) ectoderm-derived tissue cancers, B) mesoderm-derived tissue cancers, C) endoderm-derived tissue cancers, D) hormone-associated cancers, E) smoking-associated cancers, F) infectious agent-associated cancers.

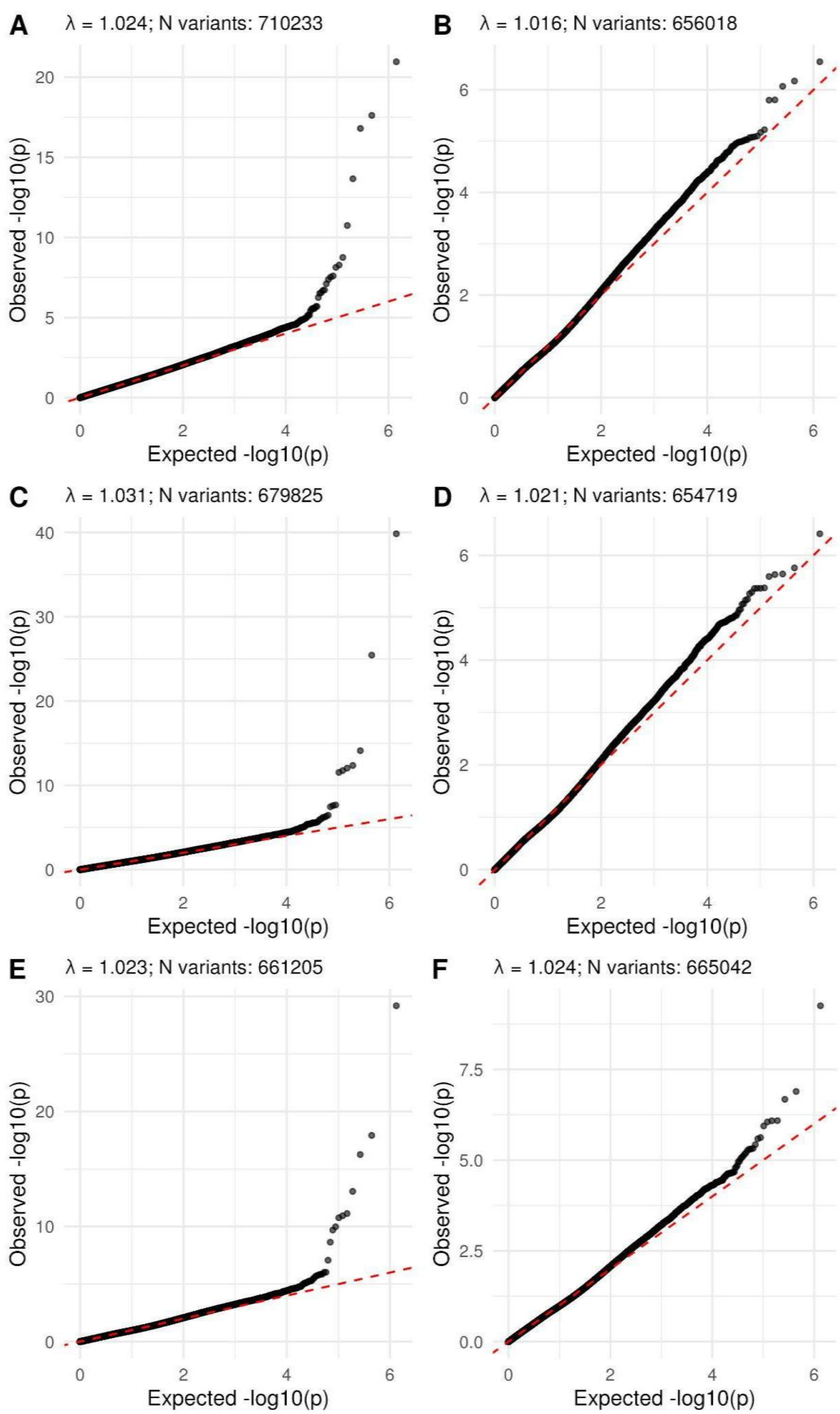

Figure S10. QQ plots with genomic inflation factors (λ) values for meta-analysis of SAIGE single variant tests. A) ectoderm-derived tissue cancers, B) endoderm-derived tissue cancers, C) hormone-associated cancers, D) infectious agent-associated cancers, E) mesoderm-derived tissue cancers, F) smoking-associated cancers.

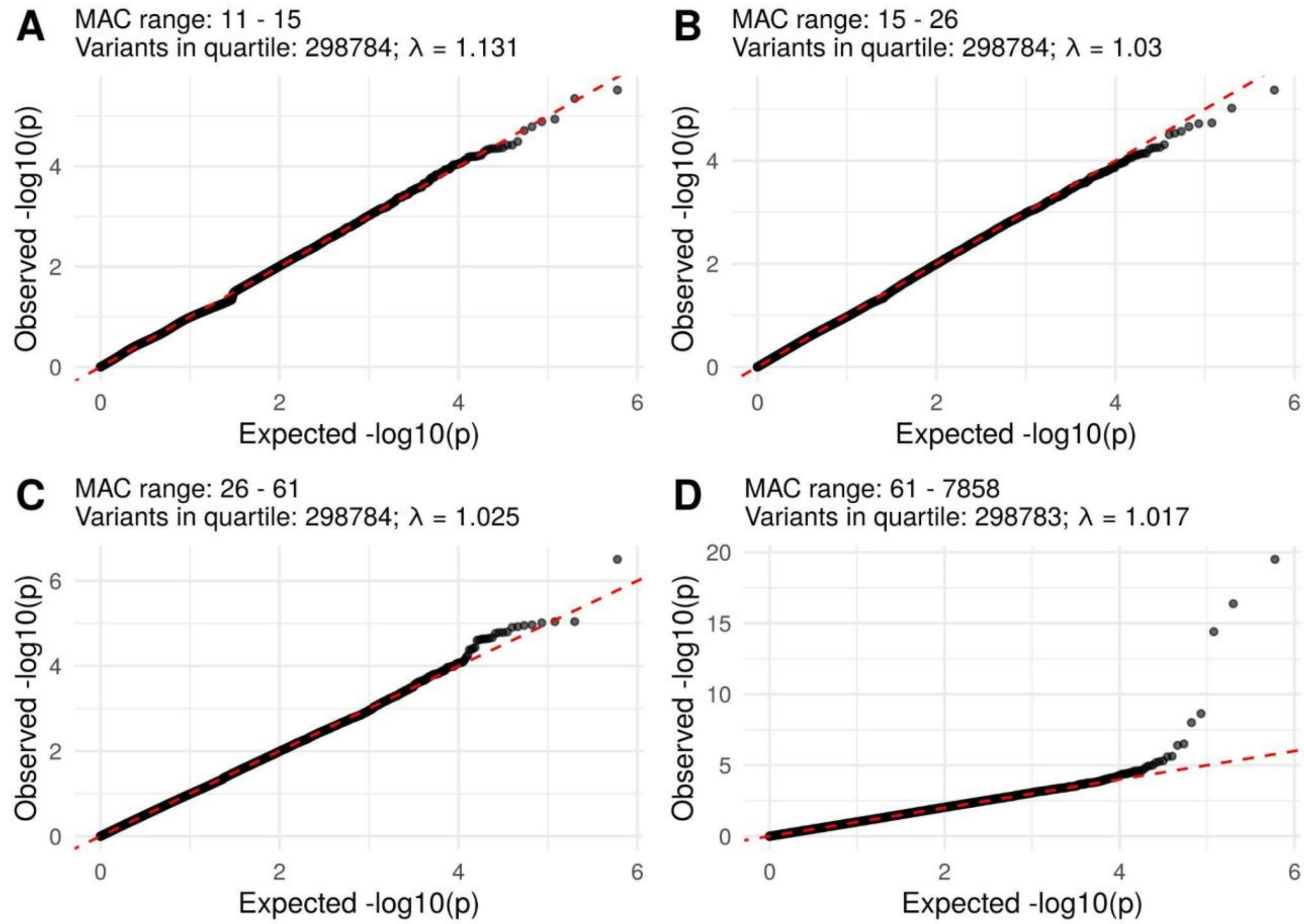

Figure S11. QQ plots with genomic inflation factors (λ) values for UKB SAIGE single variant tests of ectoderm-derived tissue cancer group by MAC quartile. A) 1st MAC quartile, B) 2nd MAC quartile, C) 3rd MAC quartile, D) 4th MAC quartile.

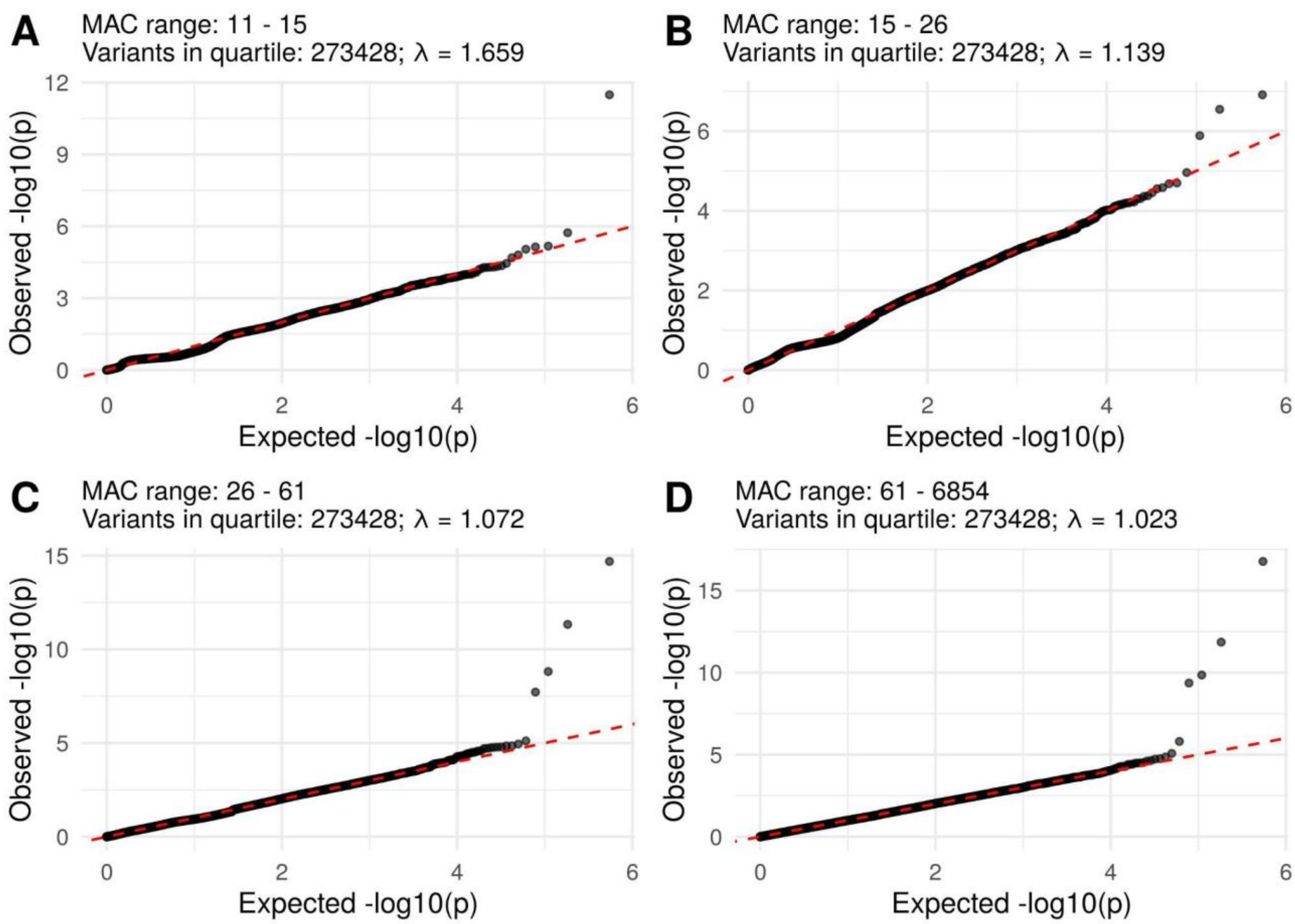

Figure S12. QQ plots with genomic inflation factors (λ) values for UKB SAIGE single variant tests of mesoderm-derived tissue cancer group by MAC quartile. A) 1st MAC quartile, B) 2nd MAC quartile, C) 3rd MAC quartile, D) 4th MAC quartile.

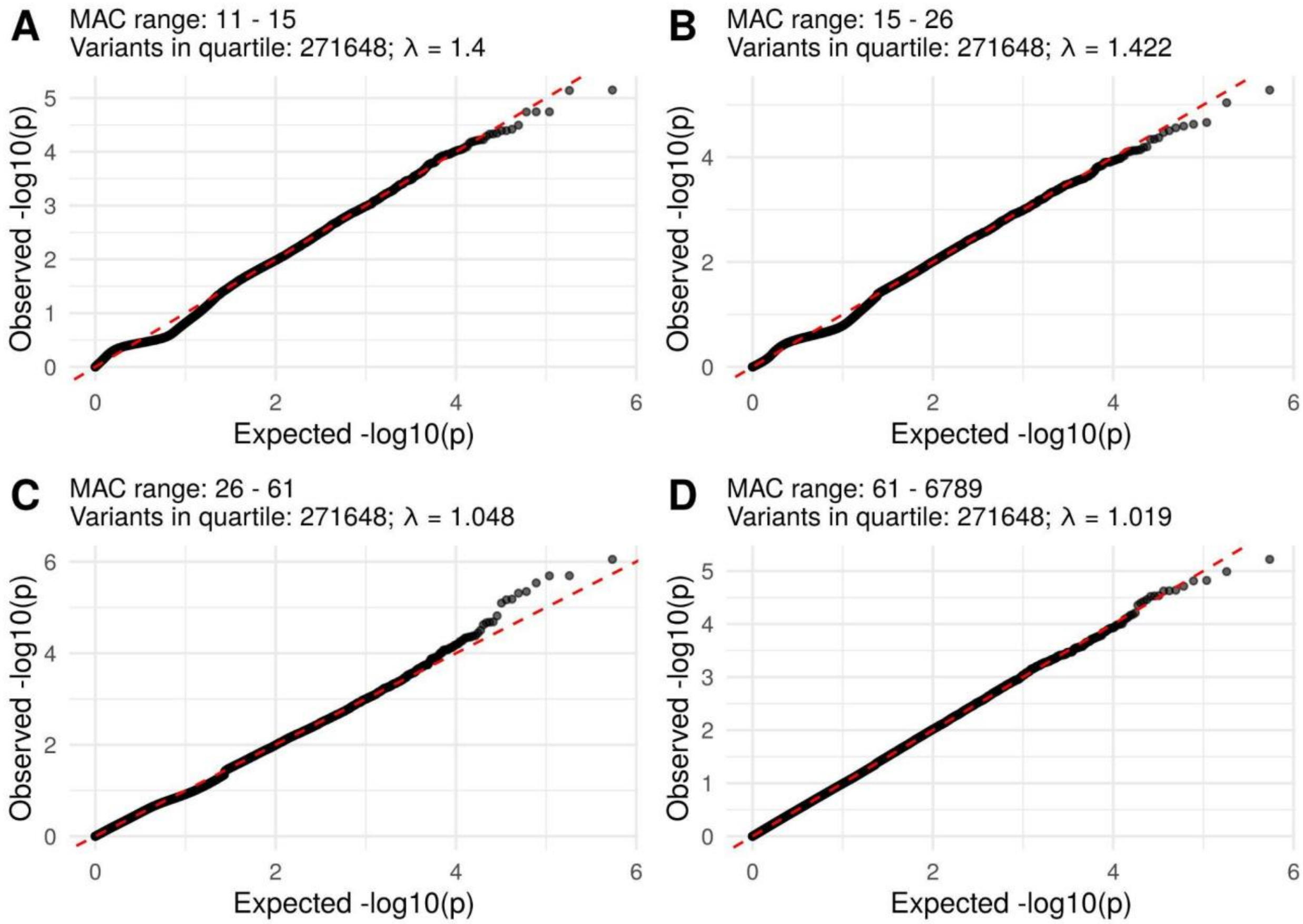

Figure S13. QQ plots with genomic inflation factors (λ) values for UKB SAIGE single variant tests of endoderm-derived tissue cancer group by MAC quartile. A) 1st MAC quartile, B) 2nd MAC quartile, C) 3rd MAC quartile, D) 4th MAC quartile.

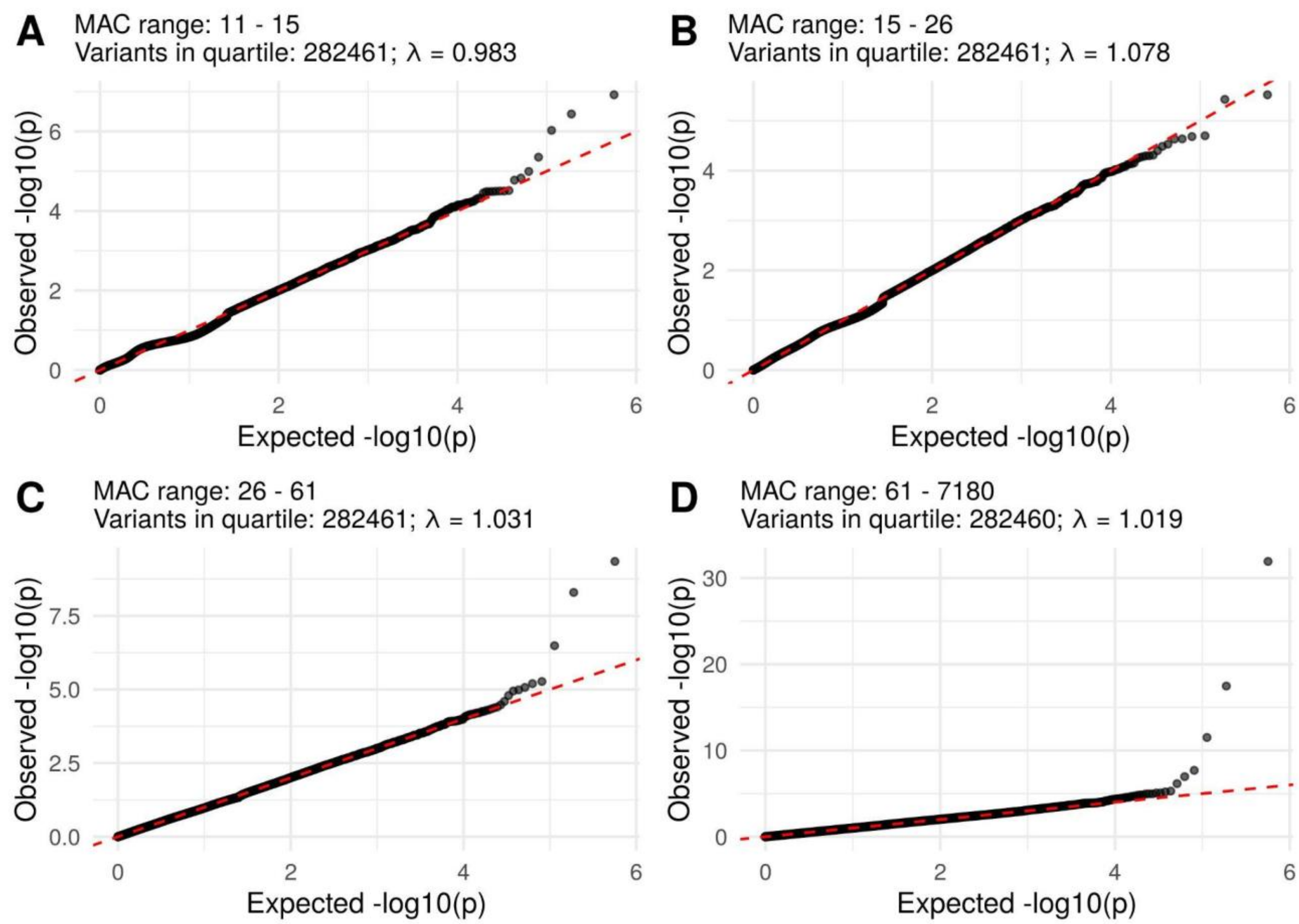

Figure S14. QQ plots with genomic inflation factors (λ) values for UKB SAIGE single variant tests of hormone-associated cancer group by MAC quartile. A) 1st MAC quartile, B) 2nd MAC quartile, C) 3rd MAC quartile, D) 4th MAC quartile.

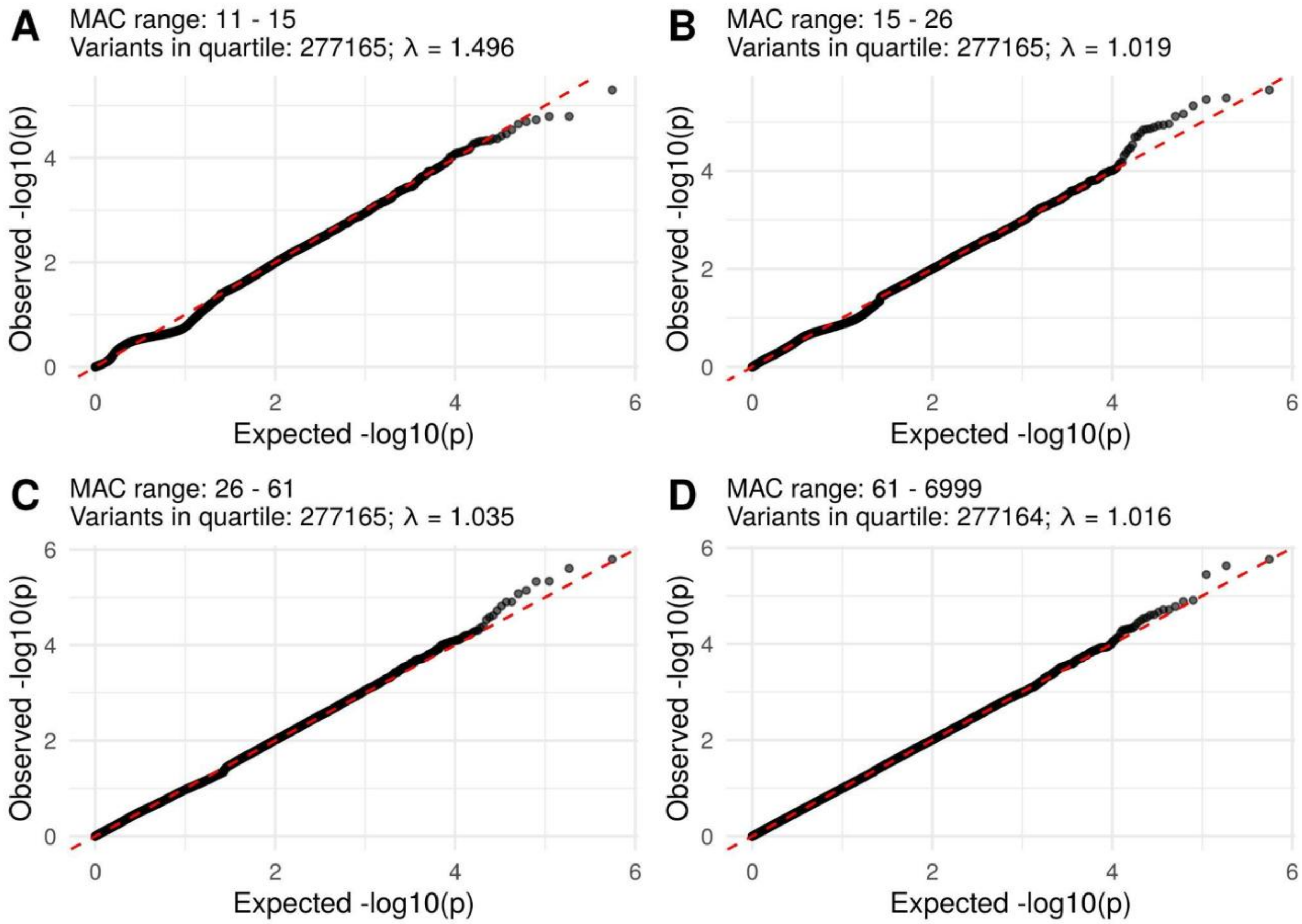

Figure S15. QQ plots with genomic inflation factors (λ) values for UKB SAIGE single variant tests of smoking-associated cancer group by MAC quartile. A) 1st MAC quartile, B) 2nd MAC quartile, C) 3rd MAC quartile, D) 4th MAC quartile.

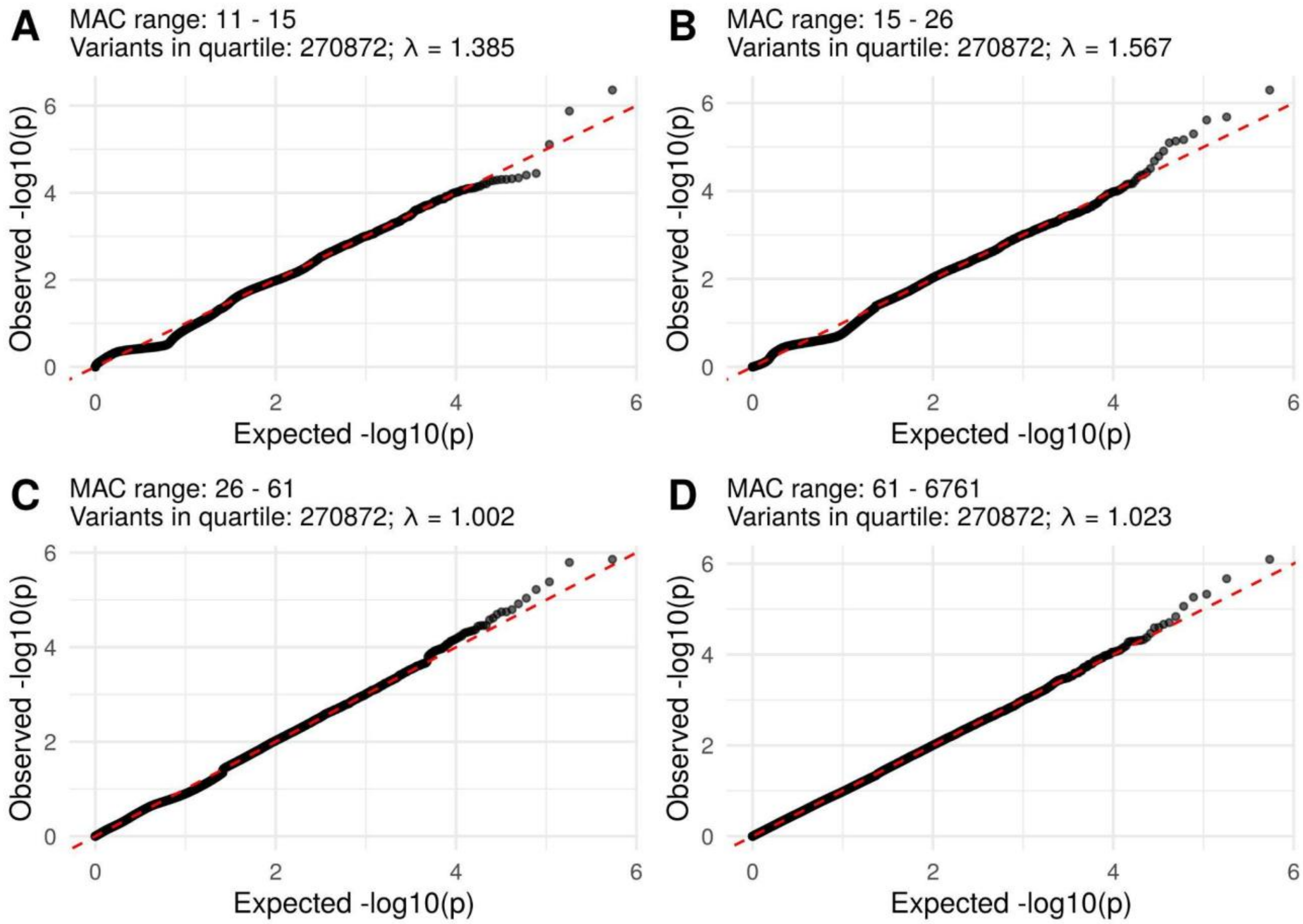

Figure S16. QQ plots with genomic inflation factors (λ) values for UKB SAIGE single variant tests of infectious agent-associated cancer group by MAC quartile. A) 1st MAC quartile, B) 2nd MAC quartile, C) 3rd MAC quartile, D) 4th MAC quartile.

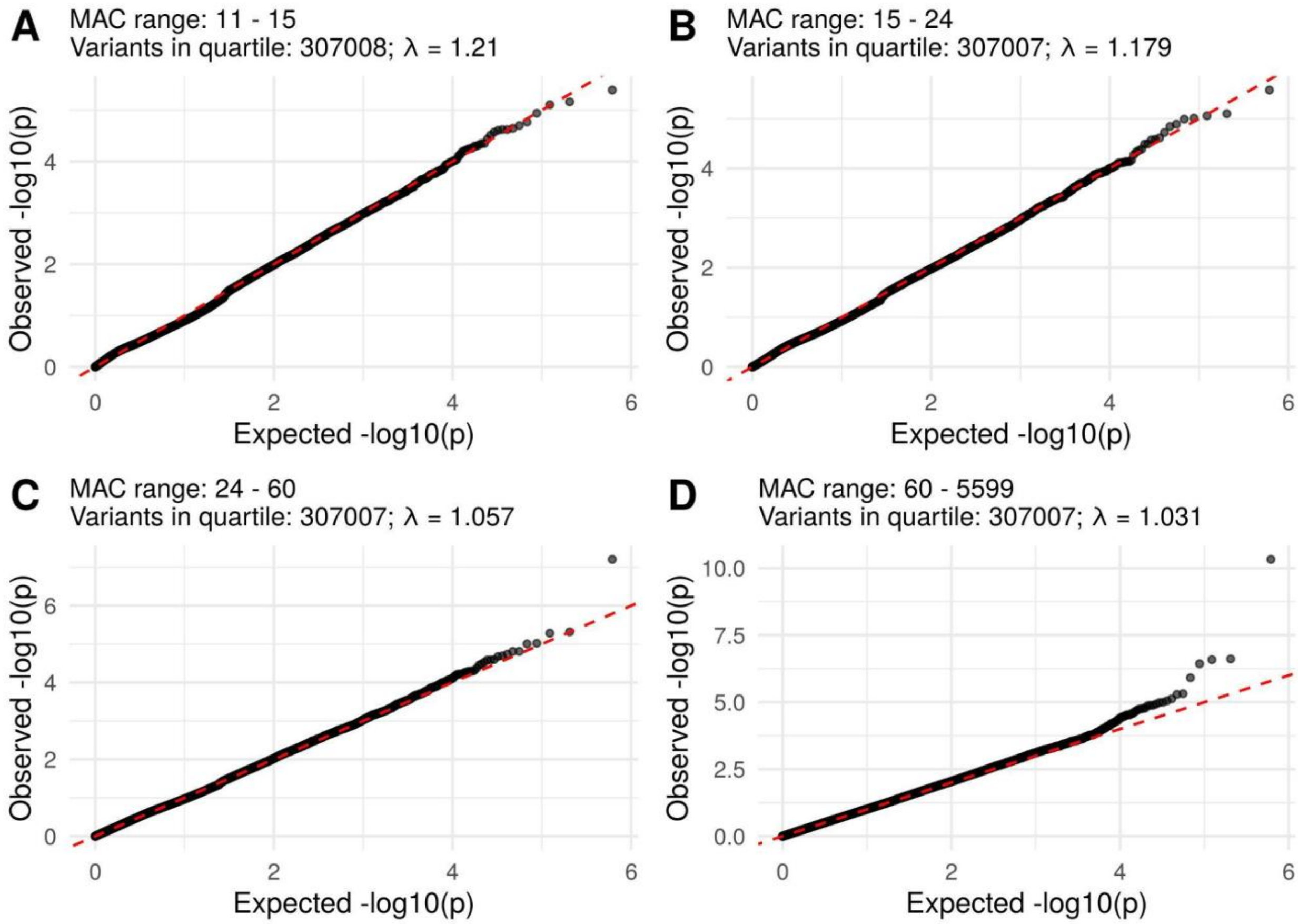

Figure S17. QQ plots with genomic inflation factors (λ) values for AoU SAIGE single variant tests of ectoderm-derived tissue cancer group by MAC quartile. A) 1st MAC quartile, B) 2nd MAC quartile, C) 3rd MAC quartile, D) 4th MAC quartile.

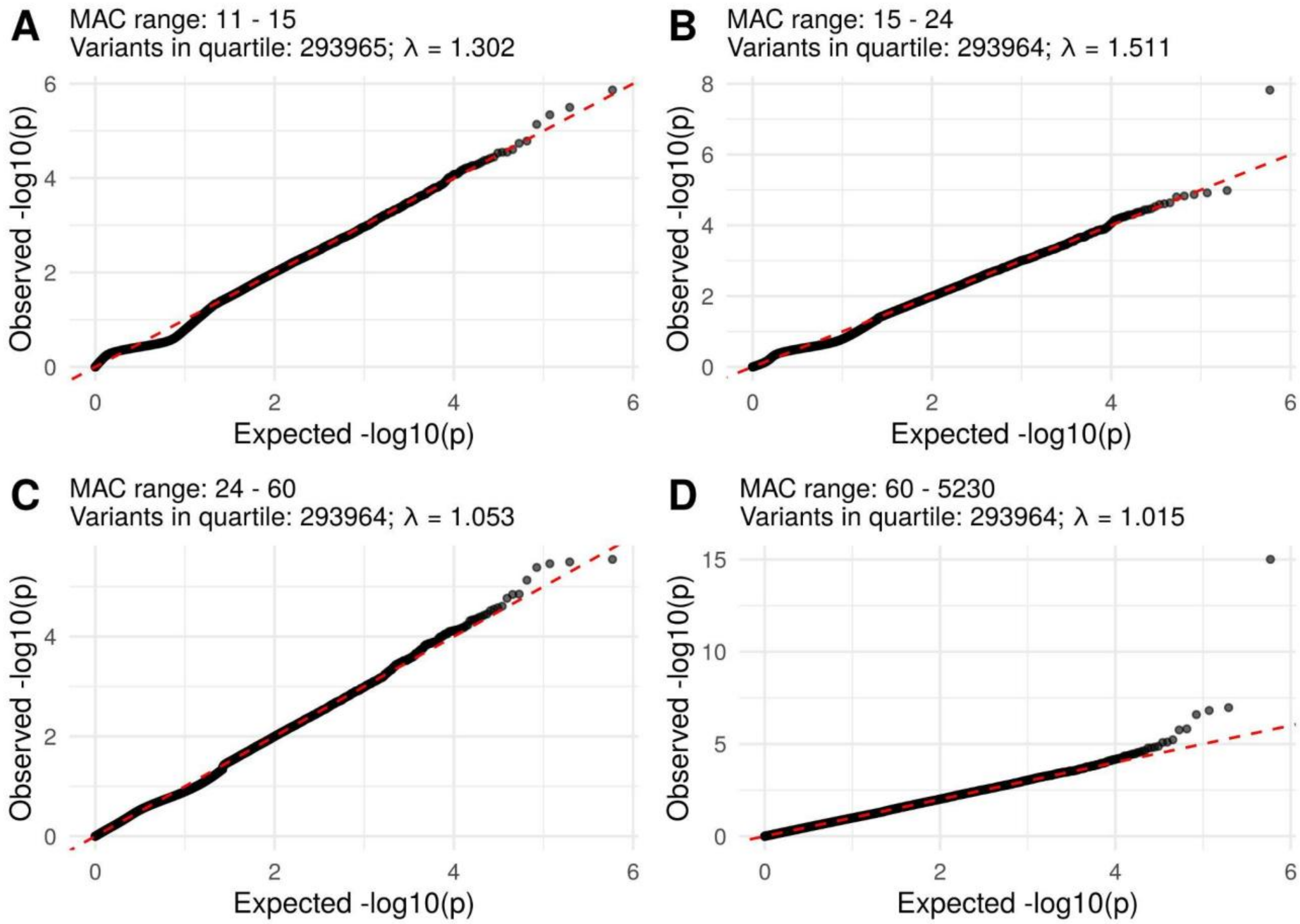

Figure S18. QQ plots with genomic inflation factors (λ) values for AoU SAIGE single variant tests of mesoderm-derived tissue cancer group by MAC quartile. A) 1st MAC quartile, B) 2nd MAC quartile, C) 3rd MAC quartile, D) 4th MAC quartile.

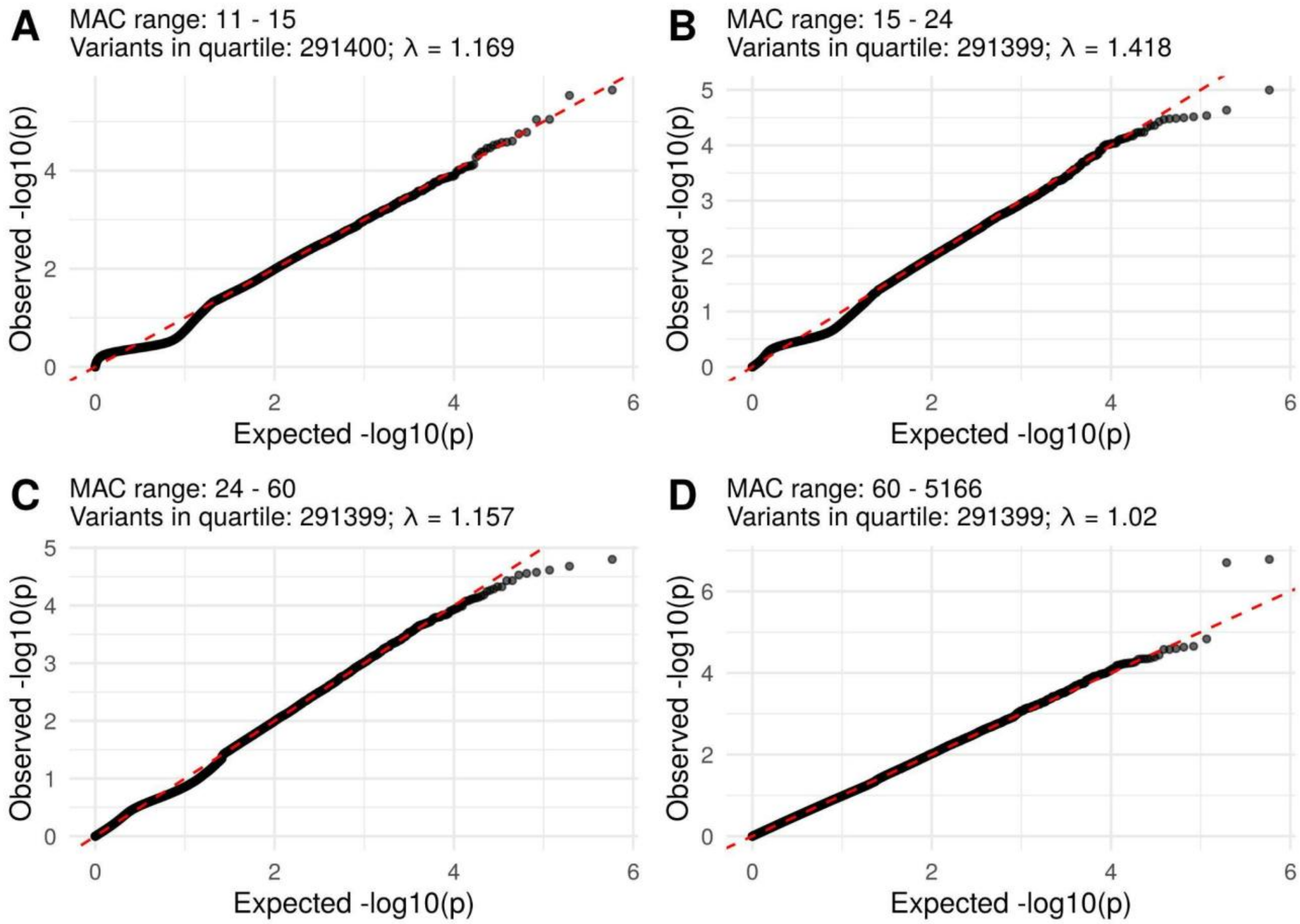

Figure S19. QQ plots with genomic inflation factors (λ) values for AoU SAIGE single variant tests of endoderm-derived tissue cancer group by MAC quartile. A) 1st MAC quartile, B) 2nd MAC quartile, C) 3rd MAC quartile, D) 4th MAC quartile.

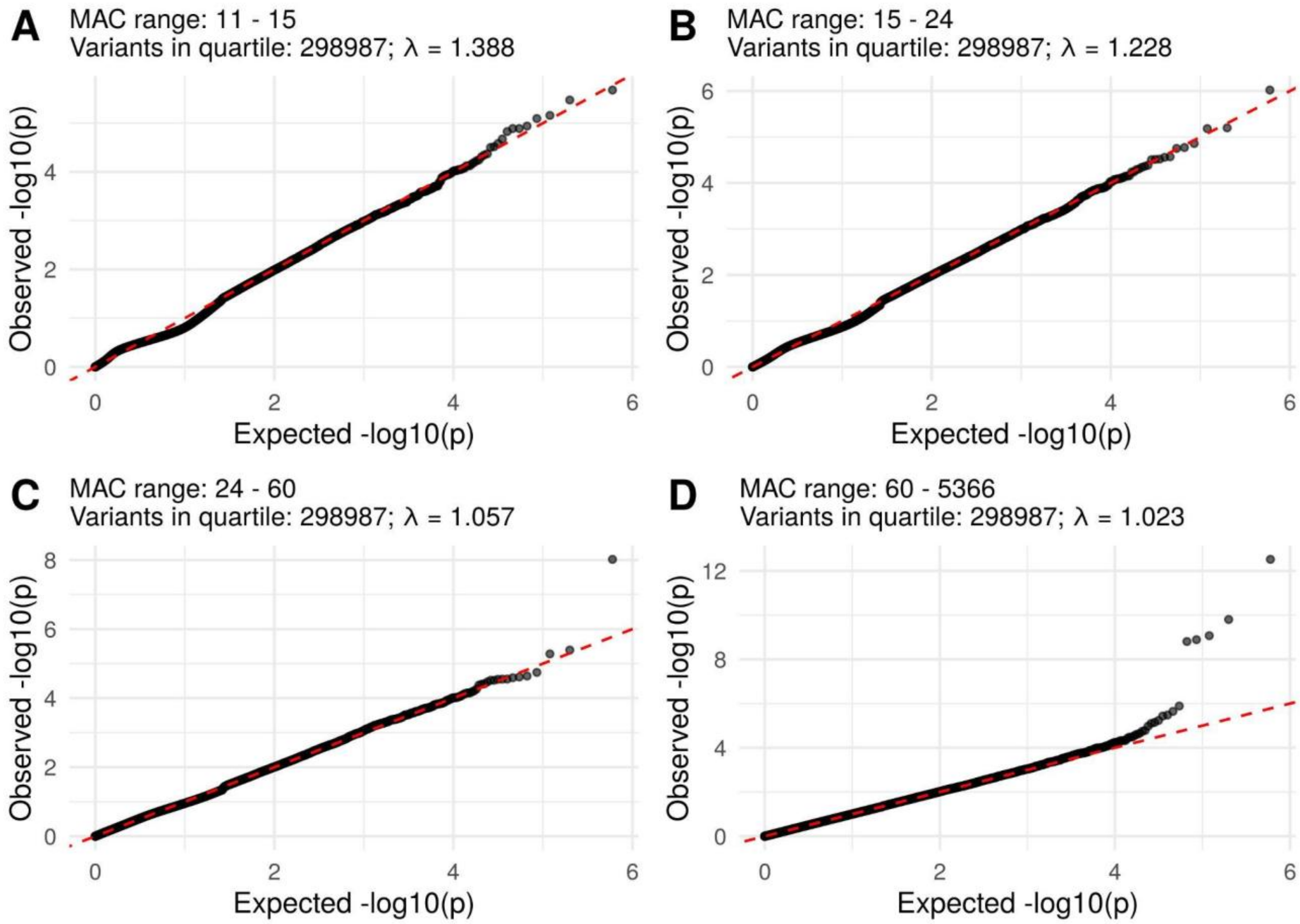

Figure S20. QQ plots with genomic inflation factors (λ) values for AoU SAIGE single variant tests of hormone-associated cancer group by MAC quartile. A) 1st MAC quartile, B) 2nd MAC quartile, C) 3rd MAC quartile, D) 4th MAC quartile.

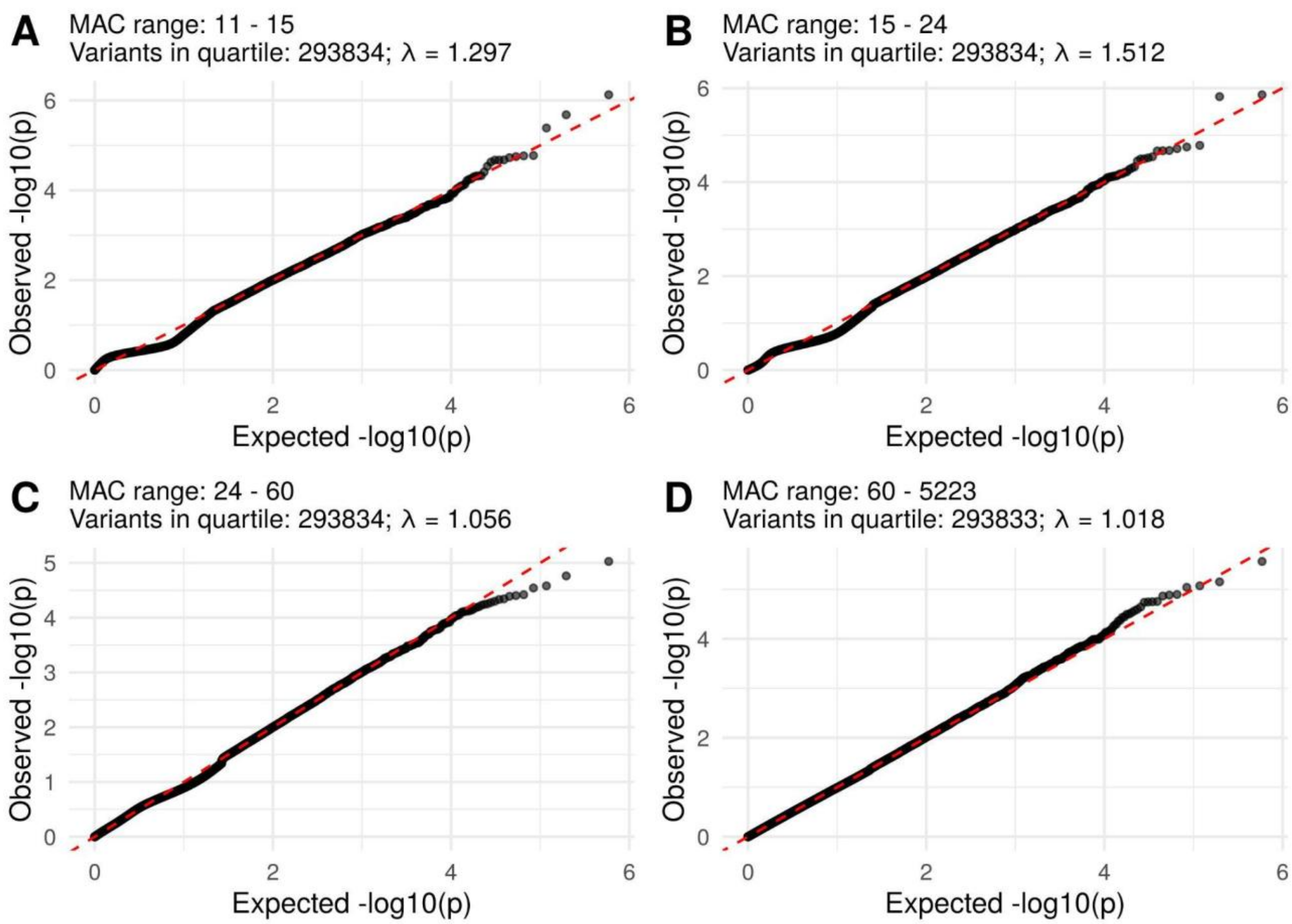

Figure S21. QQ plots with genomic inflation factors (λ) values for AoU SAIGE single variant tests of smoking-associated cancer group by MAC quartile. A) 1st MAC quartile, B) 2nd MAC quartile, C) 3rd MAC quartile, D) 4th MAC quartile.

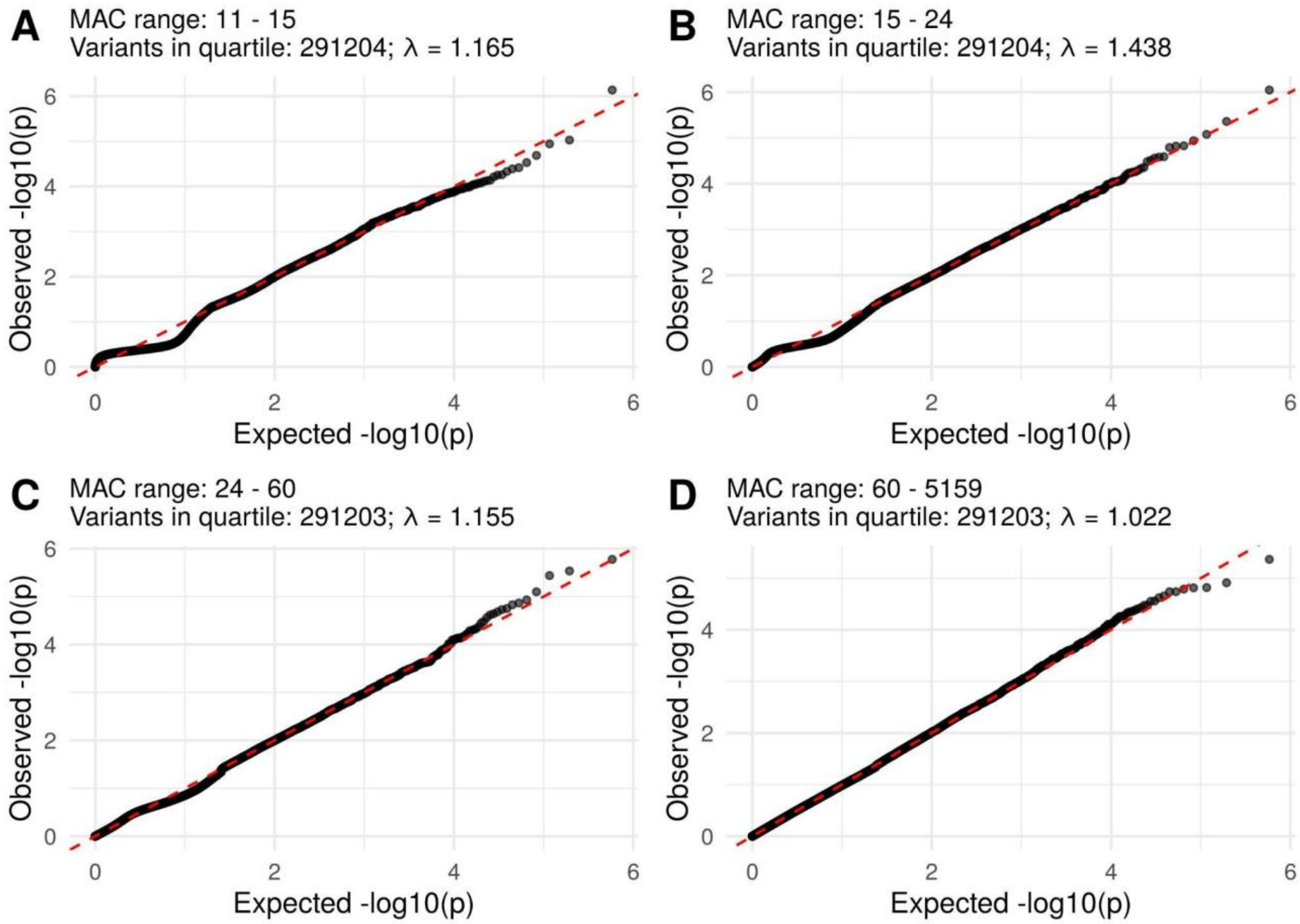

Figure S22. QQ plots with genomic inflation factors (λ) values for AoU SAIGE single variant tests of infectious agent-associated cancer group by MAC quartile. A) 1st MAC quartile, B) 2nd MAC quartile, C) 3rd MAC quartile, D) 4th MAC quartile.

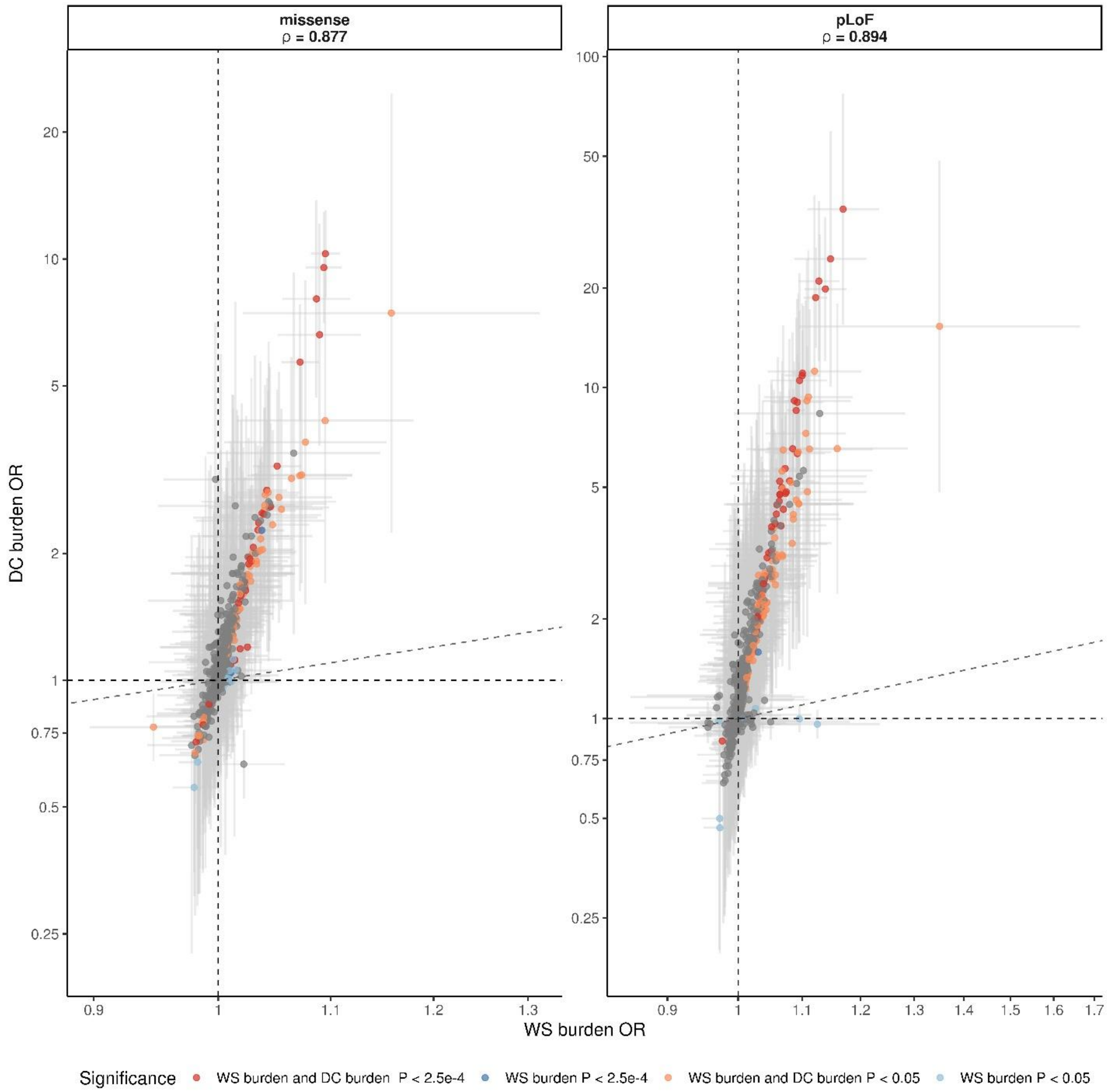

Figure S23. Overall comparison of meta-analysis OR estimates from pLoF and missense variant set MAF weighted sum (WS) and dichotomous (DC) burden tests. Points indicate OR estimates and 95% confidence intervals are indicated by vertical and horizontal grey bars. Colors indicate statistical significance groups. Overall Spearman correlations (ρ) for OR estimates from each method in pLoF or missense variant sets are indicated below each panel. Black dashed horizontal and vertical bars represent OR = 1 on each axis. The grey dashed line represents the Y = X line.

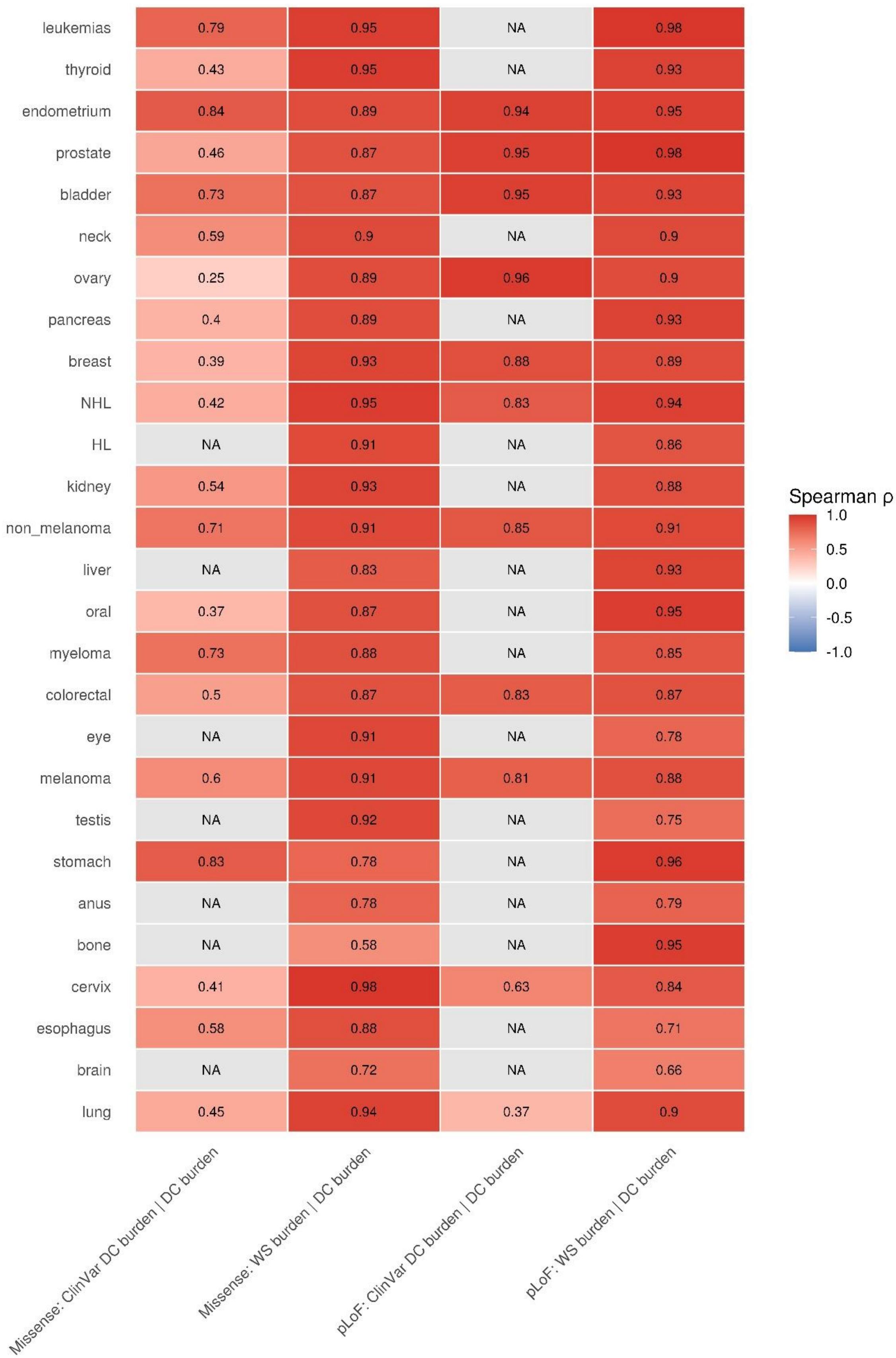
leukemias
0.79
0.95
NA
0.98
thyroid
0.43
0.95
NA
0.93
endometrium
0.84
0.89
0.94
0.95
prostate
0.46
0.87
0.95
0.98
bladder
0.73
0.87
0.95
0.93
neck
0.59
0.9
NA
0.9
ovary
0.25
0.89
0.96
0.9
pancreas
0.4
0.89
NA
0.93
breast
0.39
0.93
0.88
0.89
NHL
0.42
0.95
0.83
0.94
HL
NA
0.91
NA
0.86
kidney
0.54
0.93
NA
0.88
non_melanoma
0.71
0.91
0.85
0.91
liver
NA
0.83
NA
0.93
oral
0.37
0.87
NA
0.95
myeloma
0.73
0.88
NA
0.85
colorectal
0.5
0.87
0.83
0.87
eye
NA
0.91
NA
0.78
melanoma
0.6
0.91
0.81
0.88
testis
NA
0.92
NA
0.75
stomach
0.83
0.78
NA
0.96
anus
NA
0.78
NA
0.79
bone
NA
0.58
NA
0.95
cervix
0.41
0.98
0.63
0.84
esophagus
0.58
0.88
NA
0.71
brain
NA
0.72
NA
0.66
lung
0.45
0.94
0.37
0.9
Missense: ClinVar DC burden | DC burden
Missense: WS burden | DC burden
pLoF: ClinVar DC burden | DC burden
pLoF: WS burden | DC burden
Spearman ρ
1.0
0.5
0.0
-0.5
-1.0

Figure S24. Spearman correlations between gene-based pLoF and missense variant meta-analysis OR estimates by cancer. Cancer-specific Spearman correlations (ρ) between MAF-weighted sum (WS) and dichotomous (DC) burden tests and between DC and ClinVar variant-filtered DC burden tests. ORs were calculated separately for tests of pLoF and missense variant-based sets. We did not calculate correlations when < 10 genes had OR estimates available for a given cancer.

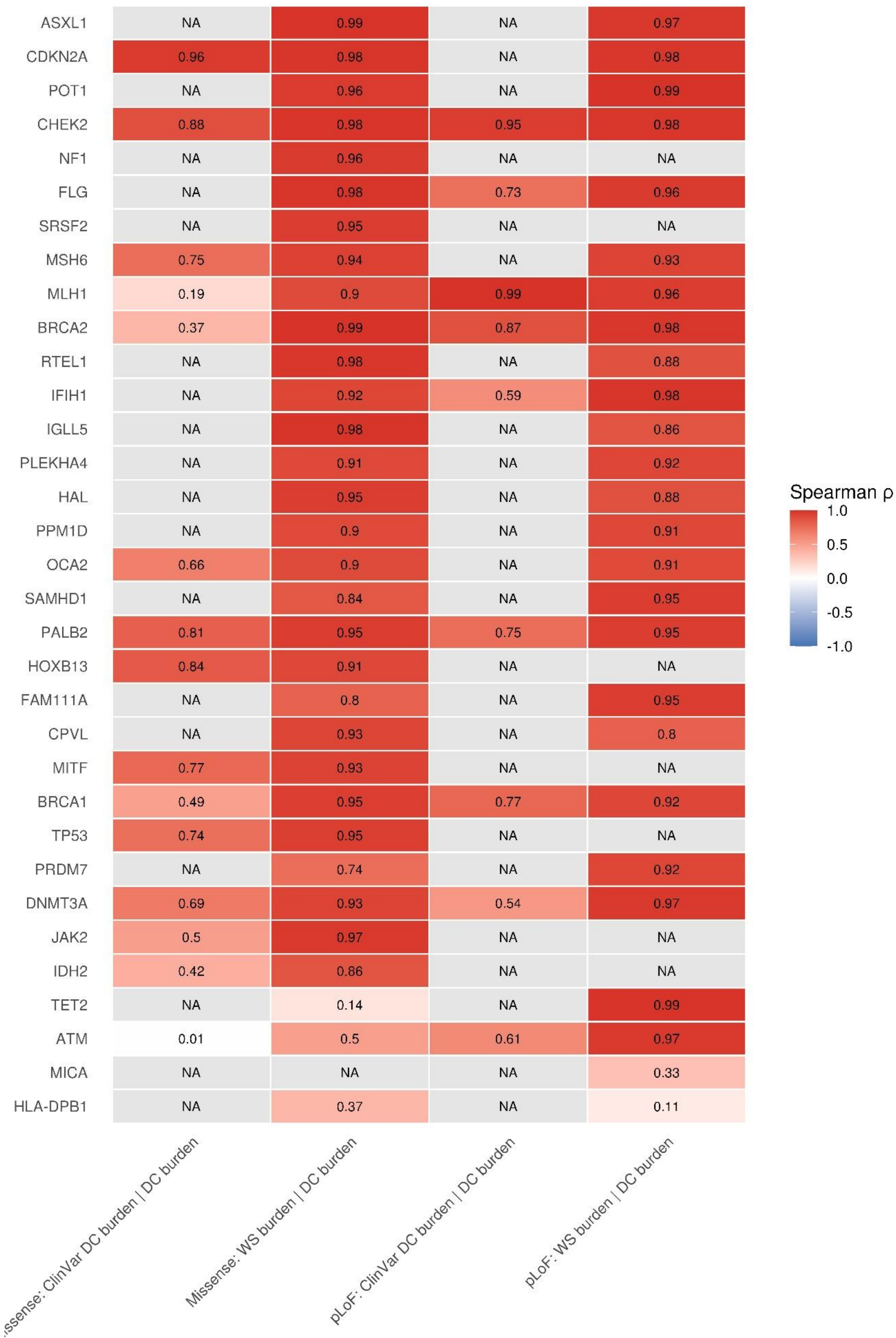

ASXL1 NA 0.99 NA 0.97
CDKN2A 0.96 0.98 NA 0.98
POT1 NA 0.96 NA 0.99
CHEK2 0.88 0.98 0.95 0.98
NF1 NA 0.96 NA NA
FLG NA 0.98 0.73 0.96
SRSF2 NA 0.95 NA NA
MSH6 0.75 0.94 NA 0.93
MLH1 0.19 0.9 0.99 0.96
BRCA2 0.37 0.99 0.87 0.98
RTEL1 NA 0.98 NA 0.88
IFIH1 NA 0.92 0.59 0.98
IGLL5 NA 0.98 NA 0.86
PLEKHA4 NA 0.91 NA 0.92
HAL NA 0.95 NA 0.88
PPM1D NA 0.9 NA 0.91
OCA2 0.66 0.9 NA 0.91
SAMHD1 NA 0.84 NA 0.95
PALB2 0.81 0.95 0.75 0.95
HOXB13 0.84 0.91 NA NA
FAM111A NA 0.8 NA 0.95
CPVL NA 0.93 NA 0.8
MITF 0.77 0.93 NA NA
BRCA1 0.49 0.95 0.77 0.92
TP53 0.74 0.95 NA NA
PRDM7 NA 0.74 NA 0.92
DNMT3A 0.69 0.93 0.54 0.97
JAK2 0.5 0.97 NA NA
IDH2 0.42 0.86 NA NA
TET2 NA 0.14 NA 0.99
ATM 0.01 0.5 0.61 0.97
MICA NA NA NA 0.33
HLA-DPB1 NA 0.37 NA 0.11
ssense: ClinVar DC burden | DC burden
Missense: WS burden | DC burden
pLoF: ClinVar DC burden | DC burden
pLoF: WS burden | DC burden
Spearman ρ
1.0
0.5
0.0
-0.5
-1.0

Figure S25. Spearman correlations between gene-based pLoF and missense variant meta-analysis OR estimates by gene. Gene-specific Spearman correlations (ρ) between MAF-weighted sum (WS) and dichotomous (DC) burden tests and between DC and ClinVar variant-filtered DC burden tests. ORs were calculated separately for tests of pLoF and missense variant-based sets. We did not calculate correlations when < 10 cancers had OR estimates available for a given gene.

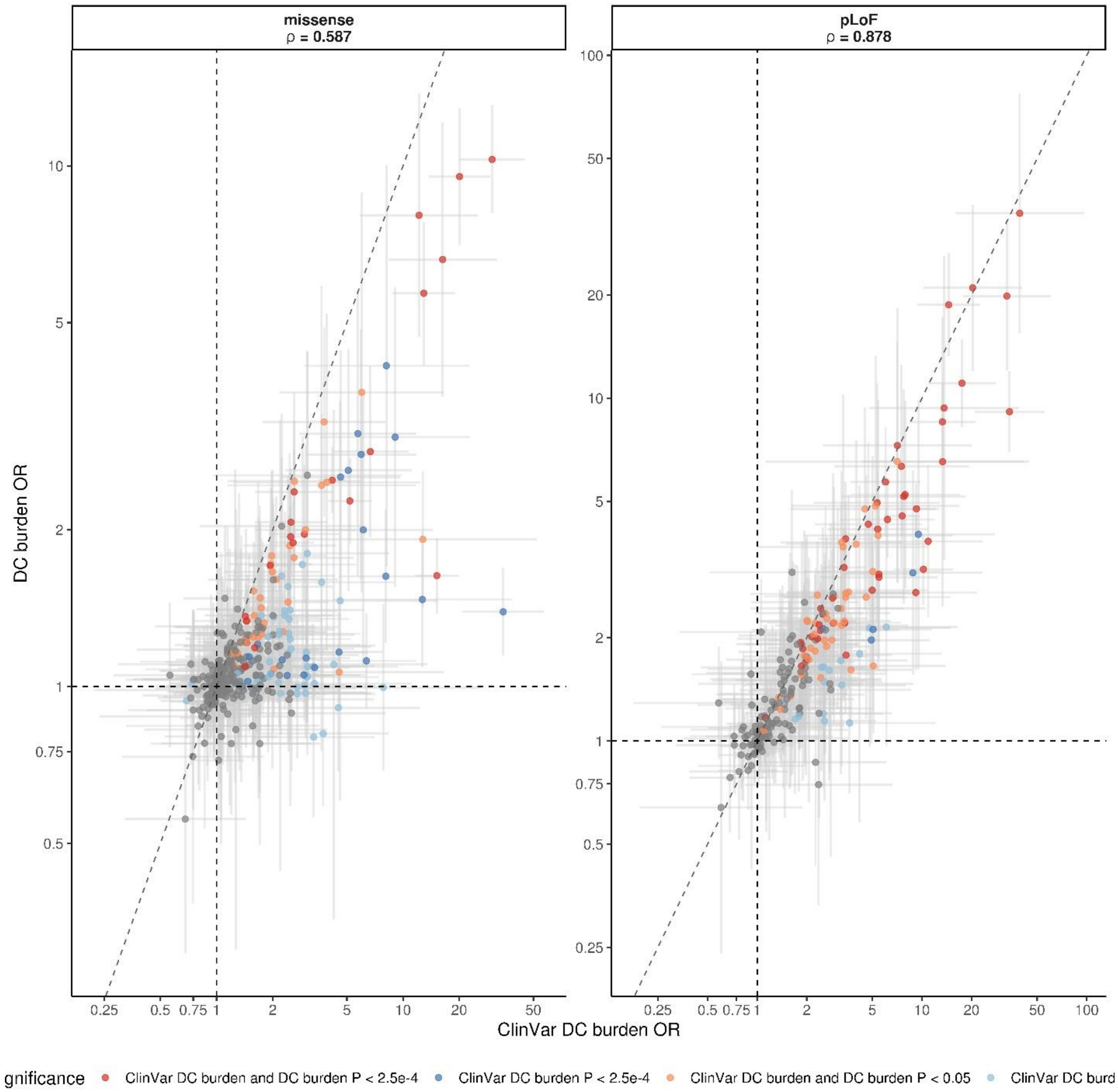

Figure S26. Overall comparison of meta-analysis OR estimates from full dichotomous (DC) and ClinVar variant-filtered DC burden tests. Points indicate OR estimates and 95% confidence intervals are indicated by vertical and horizontal grey bars. Colors indicate statistical significance groups. Overall spearman correlations (ρ) for OR estimates from each method in pLoF or missense variant sets are indicated below each panel. Black dashed horizontal and vertical bars represent OR = 1 on each axis. The grey dashed line represents the Y = X line.

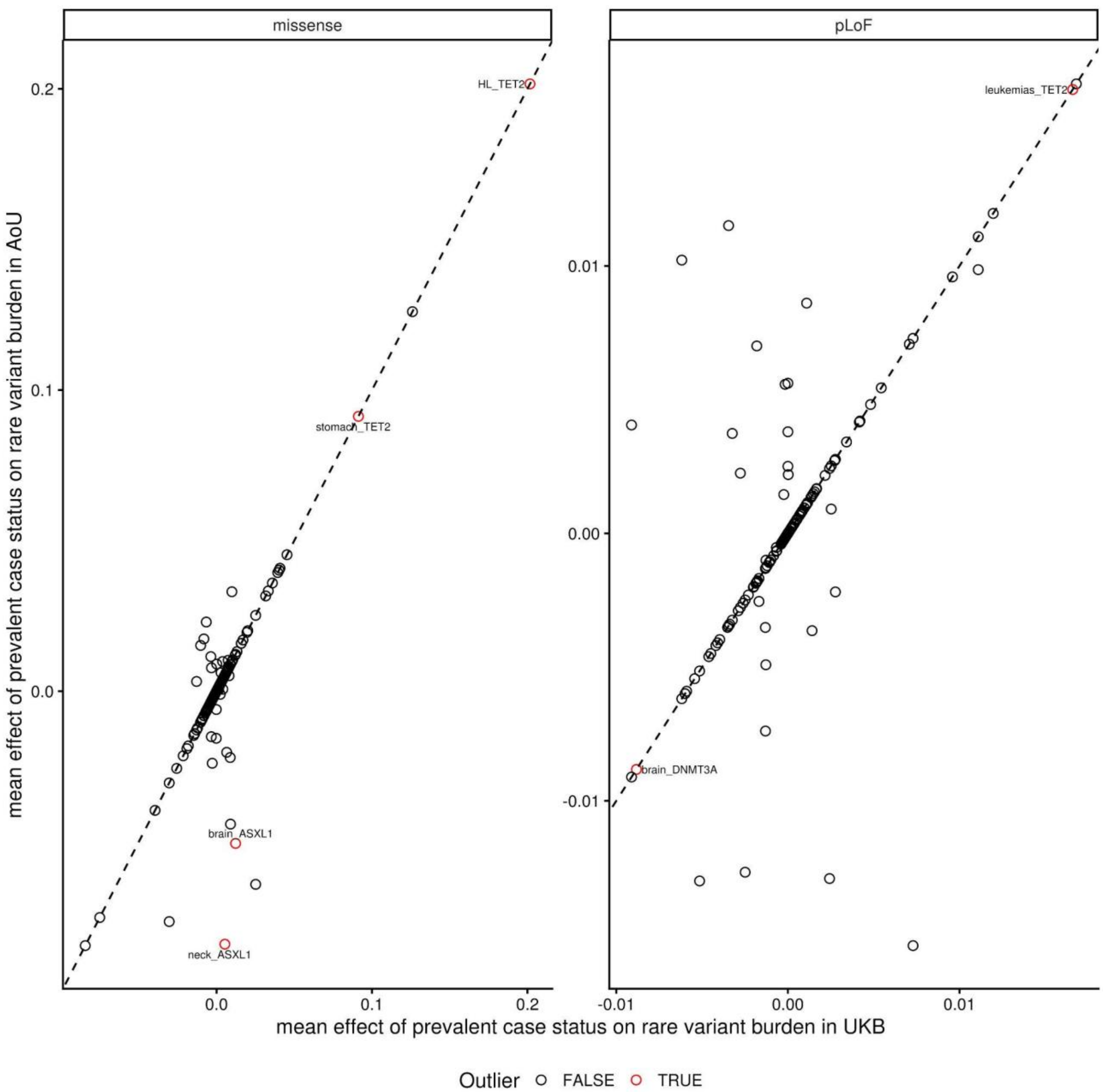

Figure S27. Results from linear regression for the effect of prevalent case status on rare variant burden in CHIP-associated genes. Points represent age at DNA sample collection adjusted prevalent case status effect estimates from linear regression models for rare variant burden in CHIP-associated genes. Estimates that were >2 standard deviations from the distribution mean and had $p < 0.05$ in either cohort are labeled as outliers. The grey dashed line represents the Y = X line.

Supplemental Methods

Whole genome and whole exome sequencing variant quality control filtering criteria

In the UK Biobank (UKB) cohort whole exome sequencing (WES) data, variants were included if they had at least 90% of genotypes with read depths DP of at least 10 for the full cohort and missingness rates ≤1% and a minor allele count (MAC) > 0 in our full analytic sample[1]. In the All of Us Research Program (AoU) cohort whole genome sequencing (WGS), variants were included if they had a minor allele count (MAC) > 0 in our full analytic sample and met the standard QC criteria defined by AoU: a genotype with genotype quality (GQ) ≥ 20, depth (DP) ≥ 10, and allele balance (AB) for heterozygotes ≥ 0.2 for the variant, excess heterozygosity Phred-scaled *p*-value < 54.69, an overall variant quality (QUAL) ≥ 60 for SNVs and ≥ 69 for short insertion or deletions (indels), and fewer than 100 alternate alleles[2]. Final variant counts are listed in Table S7.

Assessment of modeling assumptions in cancer group and single cancer analyses

To assess evidence of systematic inflation of test statistics caused by uncontrolled confounding in the cancer group analyses, we created quantile-quantile (QQ) plots and calculated genomic inflation factors (λ). λ values for cancer group analyses were calculated using the standard comparison of median of burden test statistics in the observed and expected distributions. For gene-based analyses of cancer groups in each cohort and the meta-analysis, we created QQ plots and calculated λ for the burden test results from the three primary annotation-based cRV sets (pLoF, missense, and synonymous; Figures S1 – S9). For gene-based tests of potential pleiotropic genes across individual cancers and small cancer groups, we calculated λ values for each cancer analysis for each of the three primary annotation-based cRV sets separately in each cohort. Because a smaller number of genes were tested in these analyses, λ values for the individual cancer analyses were calculated by regressing the observed and expected burden test statistics with linear regression (Tables S4 & S5). For single cRV test meta-analysis results, we created standard QQ plots for each cancer group (Figure S10). For cohort-specific cancer group single rare cRV tests, we stratified QQ plots and λ calculations by approximate MAC quartiles (Figures S11 – S22).

Assessment of the influence of somatic mutations in clonal hematopoiesis of indeterminate potential (CHIP) genes

To assess the potential influence of somatic mutations on our results, we fit linear regression models to detect associations between the number of minor alleles in a pLoF or missense cRV set in genes associated with CHIP and timing of diagnoses (prevalent vs. incident). We performed these additional analyses for any CHIP-associated genes (*ASXL1, DNMT3A, JAK2, PPM1D, SF3B1, SRSF2, TET2,* and *TP53)*[3] that were also significant in at least one of our cancer group analyses. We used the earliest date of diagnosis reported and the date of DNA collection for each case to define prevalent cases as those who were diagnosed on or before the date when their DNA was collected. All linear regression models were adjusted for age at DNA sample collection. Results of these sensitivity analyses are presented in Figure S27.

Supplemental References

1. UK Biobank (2020). UK Biobank - Exome Data Release FAQs / December 2020. https://www.ukbiobank.ac.uk/media/cfulxh52/uk-biobank-exome-release-faq_v9-december-2020.pdf.

2. All of Us Research Program (2025). All of Us Genomic Quality Report. User Support. https://support.researchallofus.org/hc/en-us/articles/29390274413716-All-of-Us-Genomic-Quality-Report.

3. Marnell, C.S., Bick, A., and Natarajan, P. (2021). Clonal Hematopoiesis of Indeterminate Potential (CHIP): Linking Somatic Mutations, Hematopoiesis, Chronic Inflammation and Cardiovascular Disease. J. Mol. Cell. Cardiol. *161*, 98–105. https://doi.org/10.1016/j.yjmcc.2021.07.004.
